# Greenhouse climate shapes nitrate levels, nutritional quality, and shelf life in leafy vegetables: species-specific responses

**DOI:** 10.3389/fpls.2026.1806482

**Published:** 2026-05-07

**Authors:** Dimitrios Fanourakis, Oliver Körner, Theodora Makraki, George P. Spyrou, Ioannis Karavidas, Georgios Tsaniklidis, Ernst Woltering, Georgia Ntatsi

**Affiliations:** 1 Laboratory of Quality and Safety of Agricultural Products, Landscape and Environment, Department of Agriculture, School of Agricultural Sciences, Hellenic Mediterranean University, Heraklion, Greece; 2 Next-Generation Horticultural Systems, Leibniz-Institute of Vegetable and Ornamental Crops (IGZ), Grossbeeren, Germany; 3 Vegetable Production, Department of Crop Science, Agricultural University of Athens, Athens, Greece; 4 Institute of Olive Tree, Subtropical Plants and Viticulture, Hellenic Agricultural Organization ‘ELGO-Dimitra’, Heraklion, Greece; 5 Horticulture and Product Physiology, Department of Plant Sciences, Wageningen University, Wageningen, Netherlands; 6 Food and Biobased Research, Wageningen University and Research, Wageningen, Netherlands

**Keywords:** chlorophyll, greenhouse climate, lettuce, nitrate reductase, phenolics, postharvest physiology, rocket, shelf life

## Abstract

This review synthesizes current knowledge on how greenhouse environmental conditions shape nitrate accumulation, nutritional quality, and shelf-life performance in lettuce, spinach, and rocket. Preharvest climate drivers interactively regulate nitrate reduction, pigment and phenolic biosynthesis, tissue structural integrity, and the physiological mechanisms determining postharvest longevity. These environmental effects influence postharvest performance by regulating carbohydrate reserves, antioxidant capacity, membrane stability, and calcium-mediated tissue integrity, which collectively determine the ability of harvested leaves to maintain metabolic homeostasis during storage. Light intensity and temperature exert the strongest influence, regulating carbon assimilation, nitrate reductase activity, and metabolic homeostasis. Adequate, well-distributed irradiance at moderate temperatures enhances chlorophyll, carotenoids, vitamin C, and phenolics while lowering nitrate levels. In contrast, low light or excessive heat suppress nitrate reduction, dilute pigments and antioxidants, and accelerate senescence. Relative air humidity (RH, via vapor pressure deficit), irrigation regime, and nutrient balance also affect quality by modulating water relations, calcium transport, and leaf structural integrity. Mild irrigation deficit and balanced nitrogen–potassium–calcium supply promote firmer tissues, reduced nitrate accumulation, and slower yellowing, whereas overirrigation or excessive nitrogen produces succulent leaves with limited storability. CO_2_ enrichment, diffuse-light films, and targeted mineral or biostimulant inputs can refine nutritional outcomes, although their effects are context-dependent and secondary to light and temperature. Species-specific sensitivities further guide climate optimization. Lettuce is particularly sensitive to low light and high RH, spinach is vulnerable to temperature-driven pigment loss, and rocket depends heavily on light quality and nitrogen balance to maintain antioxidant capacity and limit nitrate accumulation. Overall, harmonizing multiple environmental variables, rather than maximizing individual factors, offers the most effective pathway to achieving low nitrate levels, high nutritional density, and extended shelf life in greenhouse-grown leafy vegetables.

## 1 Introduction

Preharvest conditions exert a decisive influence on the nitrate content of leafy vegetables, shaping both their nutritional profile and postharvest performance (Santamaria, 2006; Colla et al., 2018). Nitrate levels reflect the dynamic balance between nitrogen uptake, nitrate assimilation, and the metabolic capacity for nitrate reduction within the leaf (Masclaux-Daubresse et al., 2010). This balance depends on key physiological processes such as nitrate reductase (NR) activity, carbon availability, and growth rate (Andrews et al., 2013). Nitrate taken up by the roots must be reduced to nitrite and subsequently to amino acids, a sequence that requires sufficient reductants and energy derived from photosynthesis (Marschner, 2012). When photosynthetic carbon supply is limited or when NR activity is constrained, due to low light, suboptimal temperature, restricted carbohydrate status, or circadian regulation, the nitrate assimilation capacity fails to match the rate of nitrate uptake (Stitt, 1999; Lillo, 2008). At the same time, rapid leaf expansion and high nitrogen demand intensify nitrate influx through the root system (Stitt, 1999). When these opposing dynamics become misaligned, whether through rapid biomass accumulation, insufficient carbon skeletons for nitrate assimilation into amino acids, reduced enzyme activity, or developmental transitions affecting sink–source balance, plants tend to accumulate elevated nitrate concentrations (Stitt, 1999; Chen et al., 2004). This accumulation is therefore not merely a consequence of high nitrogen supply, but rather the result of imbalance between uptake and reduction, driven by underlying physiological limitations and environmental constraints.

High nitrate content carries implications for food safety, regulatory compliance, and metabolic composition, and may indirectly affect postharvest performance (Santamaria, 2006; Colla et al., 2018). European Union (EU) regulatory limits differ among leafy vegetables and may vary by both season and production system (**Table 1**) (European Commission, 2011). For lettuce, the EU sets separate maximum nitrate limits for greenhouse (protected) versus open-field cultivation, recognizing that winter conditions inside greenhouses, characterized by low solar radiation and short photoperiods, substantially reduce NR activity and promote nitrate accumulation (European Commission, 2011; Colla et al., 2018). Consequently, winter thresholds for protected cultivation are higher than summer thresholds, while open-field limits are generally lower due to greater natural light availability (Santamaria, 2006; European Commission, 2011). In contrast, for spinach the EU applies a single maximum nitrate limit of 3500 mg kg^−1^ FW to fresh product regardless of production system or season, and therefore no greenhouse- versus open-field differentiation is made (European Commission, 2011). Rocket (including both *Eruca sativa* and *Diplotaxis tenuifolia*) is also regulated under a single set of limits that applies equally to greenhouse and open-field production, reflecting the EU’s non-differentiated treatment of rocket species and production systems (European Commission, 2011). For all remaining leafy vegetables, no crop-specific EU limits have been established, despite their often substantial nitrate accumulation potential (**Supplementary Table 1**). Compared with the broader group of leafy greens listed in **Supplementary Table 1**, the four species assessed in this review generally fall within the mid- to high-nitrate range, with lettuce and rocket showing some of the highest accumulation capacities observed across leafy vegetables. Actual nitrate concentrations in lettuce, spinach, and rocket depend strongly on environmental conditions, particularly light availability, nitrogen supply, growth rate, and canopy relative air humidity (RH) (Stitt, 1999; Colla et al., 2018). Understanding how these factors interact to influence nitrate assimilation is therefore essential for producing leafy vegetables with low nitrate concentrations. High nitrate levels can reduce nutritional quality by diluting beneficial metabolites (e.g., antioxidants) and are often associated with shorter postharvest performance, whereas controlled nitrate assimilation supports higher antioxidant content and improved shelf-life behavior.

**Table 1:** Typical nitrate concentration ranges in greenhouse-grown leafy vegetables and corresponding European Union (EU) maximum allowable nitrate limits for greenhouse and open-field production across seasons.

Crop	Typical nitrate range in greenhouse production	EU limit				Notes
		GREENHOUSE		OPEN FIELD		
		Winter (1 Oct–31 Mar)	Summer (1 Apr–30 Sep)	Winter (1 Oct–31 Mar)	Summer (1 Apr–30 Sep)	
Lettuce	1500–4500 (may exceed 5000 under low light)	5000	4000	4000	3000	Butterhead tends to accumulate more nitrate than Romaine.
Spinach	2000–6000 (can reach 7000+ under low light)	3500	3500	3500	3500	Spinach often exceeds limits in winter without supplemental lighting.
Rocket (*Eruca sativa*, *Diplotaxis tenuifolia*)	*Eruca*: 3000–6000; *Diplotaxis*: 2500–5000	7000	6000	7000	6000	EU does not differentiate between greenhouse and open-field rocket, nor between *Eruca* and *Diplotaxis*.

Values are expressed as mg NO_3_^−^ kg^−1^ fresh weight (FW). Winter limits apply from 1 October to 31 March, and summer limits from 1 April to 30 September. For lettuce, the EU distinguishes protected (greenhouse) cultivation from open-field production, with season-specific limits. For spinach, a single limit applies regardless of production system or season. For rocket (both *Eruca sativa* and *Diplotaxis tenuifolia*), the same seasonal limits apply regardless of production system or species.

Lettuce (*Lactuca sativa*), spinach (*Spinacia oleracea*), and rocket (*E. sativa* and *D. tenuifolia*) are selected for this review because they represent the three most nitrate-prone leafy vegetables cultivated globally. Rocket includes two commercially important species [i.e., *Eruca* (salad rocket) and *Diplotaxis* (wild rocket)] which differ markedly in growth rate, nitrate-accumulation capacity, glucosinolate and phenolic profiles, and postharvest behavior (D’Antuono et al., 2009; Mola et al., 2022; Stajnko et al., 2022). Both are therefore considered. These crops share fast growth, shallow root architecture, and high nitrogen demand, yet differ fundamentally in NR activity, carbon–nitrogen balance, pigmentation characteristics, and shelf-life physiology (Stitt, 1999; Masclaux-Daubresse et al., 2010; Colla et al., 2018). Their contrasting responses to nitrate-related processes justify a dedicated, species-specific comparative assessment. Unlike previous syntheses that typically examine individual environmental drivers or single crop species (Santamaria, 2006; Colla et al., 2018; Bian et al., 2020), this review provides an integrative physiological perspective linking greenhouse climate regulation with nitrate metabolism, nutritional quality, and postharvest shelf life through a comparative analysis of species-specific responses.

This review focuses exclusively on greenhouse studies. Greenhouse cultivation creates microclimatic conditions that differ substantially from open-field environments due to controlled fertigation, modified radiation regimes, and regulated atmospheric conditions. These production systems often combine rapid biomass production with periods of limited carbon assimilation, making greenhouse-grown leafy vegetables particularly prone to nitrate accumulation (Stitt, 1999; Colla et al., 2018). Because open-field results cannot be reliably extrapolated to protected cultivation, restricting the evidence base to greenhouse studies ensures that conclusions remain directly applicable to commercial production systems. In addition, studies conducted in fully controlled environments such as growth chambers, plant factories with artificial lighting (PFAL), or vertical farms were not included. Although these environments allow precise manipulation of variables (e.g., light intensity, photoperiod, or continuous lighting), their extremely stable conditions and minimal environmental variability often produce responses that are highly system-specific and therefore less representative of the dynamic conditions typical of commercial greenhouse production.

**Figure 1:**
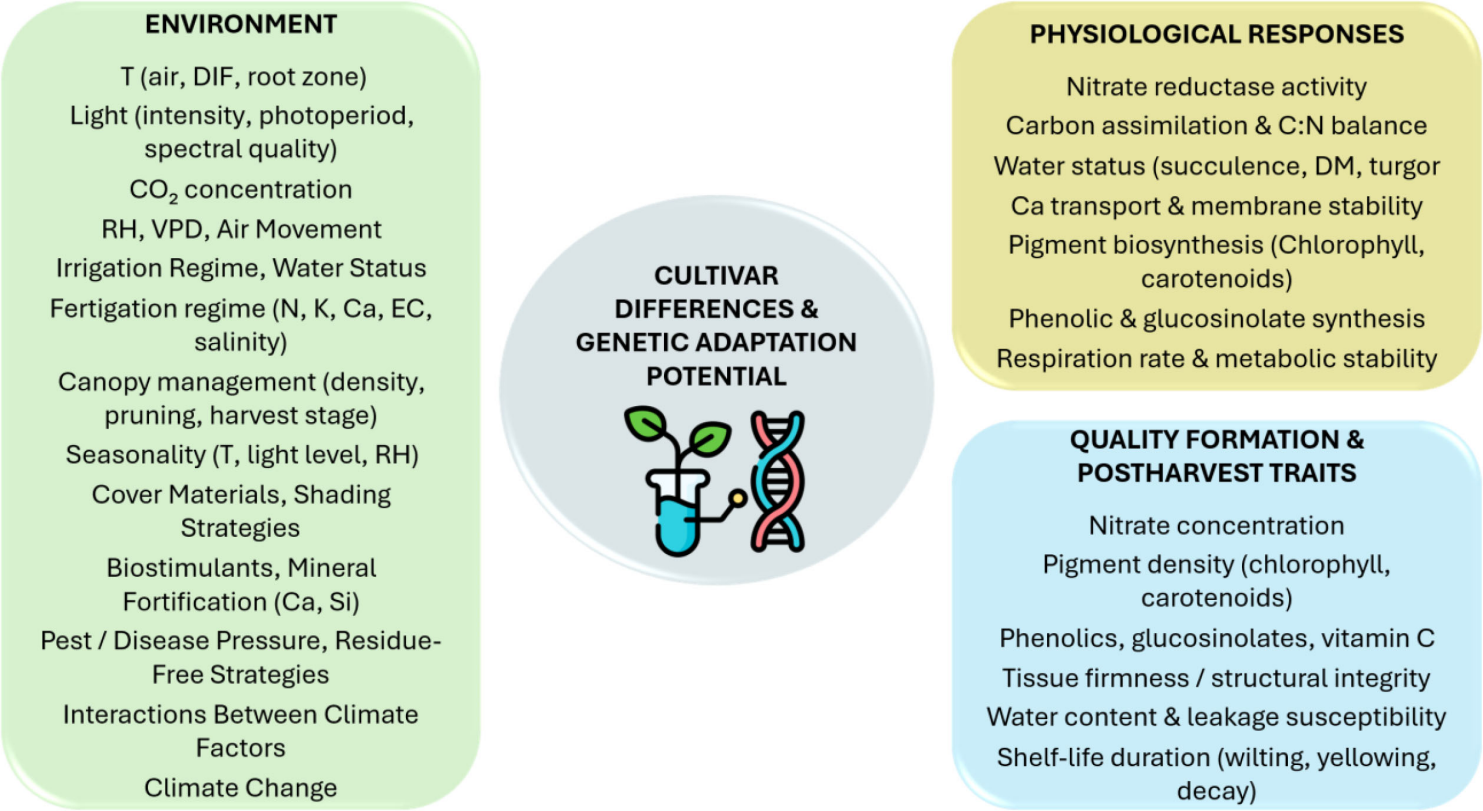
Environmental drivers of nitrate accumulation, nutritional quality, and shelf life in greenhouse leafy vegetables. C, carbon; Ca, calcium; CO_2_, carbon dioxide; DIF, day–night temperature difference; DM, dry matter; EC, electrical conductivity; K, potassium; N, nitrogen; RH, relative air humidity; Si, silicon; T, temperature; vitamin C, ascorbic acid; VPD, vapor pressure deficit.

The objective of this review is to provide an integrated, species-specific synthesis of nitrate accumulation in lettuce, spinach, and rocket under greenhouse conditions. Specifically, it aims to: (1) summarize the physiological processes governing nitrate uptake, nitrate assimilation, and accumulation; (2) compare the mechanisms underlying species-specific nitrate responses; (3) link nitrate content with postharvest outcomes, including shelf life and overall quality; and (4) identify knowledge gaps and research priorities to support sustainable, low-nitrate production in modern greenhouse systems. A conceptual overview of the environmental drivers, physiological mechanisms, and resulting quality outcomes is presented in **Figure 1**.

## 2 Concepts and metrics

### 2.1 Nutritional quality: definitions, compounds, and analytical endpoints

Nutritional quality in leafy vegetables refers to the collective presence of pigments, vitamins, minerals, antioxidants, glucosinolates, organic acids, soluble sugars, and nitrogenous compounds (Kyriacou and Rouphael, 2018; Gioia et al., 2020). These constituents determine the nutritional value, sensory attributes, and functional properties of the edible tissue (Kyriacou and Rouphael, 2018). Quantifying nutritional quality therefore requires a broad analytical framework spanning both primary and secondary metabolites, allowing a complete characterization of species-specific nutritional signatures.

Lettuce contains moderate levels of chlorophylls, carotenoids, ascorbic acid (vitamin C), phenolics, and minerals, along with small amounts of soluble sugars, which are generally low compared with other vegetables (**Table 2**) (Yang et al., 2022). Although phenolic levels are generally lower than in spinach or rocket, lettuce displays a balanced nutrient profile. Its relatively high nitrate concentration is also an important nutritional parameter (Santamaria, 2006; Colla et al., 2018). Common analytical endpoints include pigment concentration, ascorbic acid, phenolics, elemental composition, nitrate/nitrite levels, and total soluble solids (Weerakkody, 2003; Das et al., 2011; Cefola et al., 2014; Tonto et al., 2023; Shamsi et al., 2025).

**Table 2:** Key physiological, compositional, and structural traits determining nutritional quality, nitrate status, and shelf life in greenhouse-grown lettuce, spinach, and rocket.

Trait category	Lettuce	Spinach	*Eruca sativa* (salad rocket)	*Diplotaxis tenuifolia* (wild rocket)	Key references
Leaf physiology/metabolic profile	Moderate pigments; variable phenolics; moderate ascorbic acid; small amounts of soluble sugars	Very high chlorophylls; high lutein, folates, ascorbic acid; notable oxalates	High glucosinolates, phenolics, ascorbic acid; strong sulfur volatiles; high chlorophyll	Higher phenolics and glucosinolates than *Eruca*; more stable pigments; stronger aromatic profile	(Noonan, 1999; Bergquist et al., 2006; D’Antuono et al., 2009; Shohag et al., 2011; Bell et al., 2016, p. 201; Caruso et al., 2018; Yang et al., 2022; Villani et al., 2023)
Nitrate-related characteristics	Typically high NO_3_^−^ due to low NR activity	Moderate–high NO_3_^−^; efficient nitrate assimilation but still accumulates under low light	High NO_3_^−^ accumulation, especially under rapid growth and ample N supply	Lower NO_3_^−^ accumulation due to slower growth and higher NR activity	(Chen et al., 2004; Kim et al., 2006; Santamaria, 2006; Caruso et al., 2018; Villani et al., 2023)
Dominant nutritional-quality determinants	Chlorophylls, carotenoids, phenolics (low–moderate), ascorbic acid, minerals, soluble sugars	Chlorophylls, lutein, folates, ascorbic acid, Fe and Mg, phenolics, oxalates	Glucosinolates, phenolics, ascorbic acid, chlorophylls, sulfur volatiles	Higher glucosinolates, phenolics, ascorbic acid, aromatic volatiles; more pungent	(Noonan, 1999; Bergquist et al., 2006; D’Antuono et al., 2009; Shohag et al., 2011; Bell et al., 2016; Agricultural Research Service, 2021; Biondi et al., 2021; Yang et al., 2022; Villani et al., 2023)
Typical analytical endpoints	Pigments, ascorbic acid, total phenolics, minerals, soluble solids, NO_3_^−^	Pigments, lutein, ascorbic acid, folates, minerals, oxalates, phenolics, NO_3_^−^	Glucosinolates, phenolics, ascorbic acid, pigments, volatiles, minerals, NO_3_^−^	Same as *Eruca*, with consistently higher glucosinolates and phenolics	(Weerakkody, 2003; Bergquist et al., 2006; D’Antuono et al., 2009; Shohag et al., 2011; Agricultural Research Service, 2021; Martínez-Ispizua et al., 2022a; Brindisi et al., 2023; Villani et al., 2023)
Primary shelf-life limitations	Wilting, enzymatic browning, pinking, microbial spoilage	Yellowing, tissue softening, microbial growth, water loss	Very rapid wilting, yellowing, less electrolyte leakage, off-odors, microbial spoilage	Rapid yellowing but slightly slower than *Eruca*; less leakage; stronger aroma loss	(Bergquist et al., 2006; Kim et al., 2006; Nicola et al., 2010; Medina et al., 2012; Bell et al., 2016; Yang et al., 2022)
Key kinetic/end-point indicators	% weight loss, chlorophyll degradation, browning index, respiration rate, microbial counts	Moisture loss kinetics, yellowing rate, respiration, microbial load	% weight loss, chlorophyll loss, slower electrolyte leakage, volatile off-odors, acceptability scores	Similar indicators as *Eruca* but slower leakage and more stable volatiles	(Bergquist et al., 2006; Kim et al., 2006; D’Antuono et al., 2009; Nicola et al., 2010; Das et al., 2011; Medina et al., 2012; Bell et al., 2016; Brindisi et al., 2023; Tonto et al., 2023; Villani et al., 2023; Shamsi et al., 2025)
Cuticle and epidermis	Moderate cuticle; susceptible to mechanical damage	Thicker and more robust leaves; strong midrib	Very thin cuticle; high stomatal density; high transpiration	Slightly thicker cuticle than *Eruca*; lower transpiration; improved water retention	(Nicola et al., 2010; Hardin et al., 2011; Medina et al., 2012; Bell et al., 2016; Biondi et al., 2021; Chase et al., 2024; Luo et al., 2024)
Anatomical/structural factors affecting storability	Soft, delicate tissues prone to bruising and browning	Thick leaf blades, strong midrib, higher mechanical strength	Thin leaves; high surface-to-volume ratio → rapid water loss and deterioration	More robust leaf structure; slower collapse; greater mechanical integrity	(Bergquist et al., 2006; Nicola et al., 2010; Medina et al., 2012; Chase et al., 2024)
Typical storage T threshold	0–5 °C	0–5 °C	0–5 °C	0–5 °C	(Nicola et al., 2010; Medina et al., 2012; Caruso et al., 2018; Peng and Simko, 2023)
Indicative nutritional highlights (per 100 g FW)	Ascorbic acid 5–15 mg; moderate phenolics; low carotenoids	Ascorbic acid 25–50 mg; high chlorophylls; lutein-rich	Ascorbic acid 20–40 mg; high glucosinolates; moderate carotenoids	Ascorbic acid 20–40 mg; very high glucosinolates and phenolics; more persistent pigments	(Bergquist et al., 2006; D’Antuono et al., 2009; Garg and Sharma, 2014; Mola et al., 2022; Agricultural Research Service, 2024)
Overall postharvest resilience	Moderate; sensitive to browning and mechanical damage	Moderate–low; high respiration but robust tissues delay collapse	Very low; highly perishable due to thin leaves and fast respiration	Low–moderate; still perishable, but slightly better structural resilience and slower quality decline than *Eruca*	(Bergquist et al., 2006; Nicola et al., 2010; Medina et al., 2012; Peng and Simko, 2023; Chase et al., 2024)

Ascorbic acid, vitamin C; a*, red color coordinate; b*, yellow color coordinate; FW, fresh weight; NO_3_^−^, nitrate; NR, nitrate reductase; T, temperature; WC, water content.

Spinach is distinguished by high concentrations of chlorophylls, lutein, folates, ascorbic acid, and minerals such as iron and magnesium (**Table 2**) (Weerakkody, 2003; Bergquist et al., 2006; Agricultural Research Service, 2021). Its antioxidant activity is supported by abundant phenolics and flavonoids (Gil et al., 1999). Spinach also contains oxalates, which are nutritionally relevant (Noonan, 1999). Nitrate may be present at moderate to high levels depending on physiological status (Chen et al., 2004; Santamaria, 2006). Analytical endpoints include pigments, vitamins, phenolic compounds, minerals, oxalates, and nitrate (Weerakkody, 2003; Colla et al., 2018).

Rocket is characterized by high levels of glucosinolates, phenolics, ascorbic acid, and sulfur-containing volatiles that determine its pungent flavor and strong antioxidant capacity (**Table 2**) (D’Antuono et al., 2009; Mola et al., 2022). Chlorophyll concentration is typically high, giving the leaves their intense green coloration (Biondi et al., 2021). *Eruca* (salad rocket) generally accumulates more nitrate due to its rapid growth and high nitrogen demand, whereas *Diplotaxis* (wild rocket) tends to show lower nitrate levels and more stable phytochemical profiles (Santamaria, 2006; Caruso et al., 2018; Villani et al., 2023). Analytical endpoints include glucosinolate composition, phenolics, ascorbic acid, pigments, mineral elements, volatile sulfur compounds, and nitrate (Santamaria, 2006; Biondi et al., 2021).

Collectively, the nutritional quality of lettuce, spinach, and rocket comprises a broad spectrum of primary and secondary metabolites. Although nitrate constitutes an important and regulated nutritional variable, it forms only one part of a much wider biochemical matrix that differs substantially among species. Comprehensive assessment must therefore integrate multiple metabolite classes to fully represent the nutritional quality profiles of these leafy vegetables.

### 2.2 Shelf life: operational definition, kinetic endpoints, and disorder metrics

Shelf life is defined as the period during which a product retains acceptable visual, textural, nutritional, and microbial quality (Kader, 2002). In leafy vegetables, deterioration is driven by water loss, respiration, pigment degradation, enzymatic reactions, and microbial proliferation (Kader, 2002). Evaluating shelf life requires kinetic measurements and disorder metrics that capture these processes and allow clear comparison among species (Kader, 2002; Corradini, 2018). Recent analyses have emphasized how species-specific microclimate sensitivities determine postharvest performance, with environmental drivers [e.g., temperature, vapor pressure deficit (VPD), and light regime] exerting differential effects on shelf-life behavior across greenhouse vegetables (Fanourakis et al., 2025a).

Lettuce shelf life is influenced by wilting, browning, pinking, and tissue breakdown (**Table 2**) (Chase et al., 2024; Yang et al., 2024). Visual deterioration often appears as marginal browning or internal discoloration, while texture loss reflects reductions in turgor (Santamaria, 2006; Yang et al., 2024). Kinetic endpoints for lettuce include respiration rate, chlorophyll degradation, browning indices, tissue strength, and microbial counts (Cefola et al., 2014; Tonto et al., 2023).

Spinach has a short shelf life characterized by rapid yellowing, tissue softening, and microbial spoilage (**Table 2**) (Kader, 2002; Medina et al., 2012). High respiration rate and leaf fragility contribute to its rapid decline (Medina et al., 2012). Spinach quality assessment typically includes measurements of moisture loss, pigment degradation, texture changes, respiration kinetics, and microbial development (Weerakkody, 2003; Colla et al., 2018).

Rocket exhibits very rapid postharvest deterioration due to its thin leaves, high water content (WC), and elevated respiration rate (**Table 2**) (Nicola et al., 2010; Bell et al., 2016). *Eruca* (salad rocket) declines particularly quickly, showing fast wilting, yellowing, electrolyte leakage, and the development of pungent off-odors, often accompanied by microbial growth (Nicola et al., 2010; Bell et al., 2016). *Diplotaxis* (wild rocket) displays similar symptoms but typically at a slower pace owing to its slightly thicker leaves and more resilient tissue structure (Nicola et al., 2010; Bell et al., 2016). Shelf-life evaluation in both species includes measurements of water-loss kinetics, chlorophyll degradation, electrolyte leakage, volatile-aroma changes, microbial load, and overall sensory acceptability (Nicola et al., 2010; Bell et al., 2016).

Overall, shelf life in lettuce, spinach, and rocket is defined by species-specific deterioration pathways and rates. Despite shared mechanisms such as respiration and pigment degradation, the magnitude and sequence of quality loss differ among the three leafy vegetables, with the metabolic profile influencing the manifestation of damage (e.g., browning) rather than susceptibility to mechanical injury. This necessitates species-tailored shelf-life metrics and evaluation criteria.

### 2.3 Crop biology relevant to postharvest behavior

Species-specific biological traits (e.g., such as leaf anatomy, cuticle development, stomatal density, respiration rate, pigment composition, and nitrogen metabolism) strongly influence postharvest behavior (Kader, 2002; Corradini, 2018). These intrinsic characteristics determine how fast water is lost, how pigments and antioxidants are retained, and how tissues respond to stress after harvest. Nitrate content, a central focus of this review, is also rooted in these biological features and contributes to species differences.

Lettuce exhibits moderate leaf thickness, relatively limited cuticular protection, and a metabolite composition that renders it susceptible to oxidative discoloration under mechanical stress (**Table 2**) (Bergquist et al., 2006; Chase et al., 2024; Luo et al., 2024). Its comparatively low NR capacity contributes to higher internal nitrate pools, a trait relevant to nutritional labeling and quality assessment (Chen et al., 2004; Santamaria, 2006). Postharvest behavior is closely linked to its anatomy and moderate respiration rate, which together shape susceptibility to browning and wilting.

Spinach has thicker, more robust leaves with higher concentrations of chlorophylls, carotenoids, and folates (**Table 2**) (Kader, 2002; Gioia et al., 2020; Agricultural Research Service, 2021). Its tissue structure confers better resistance to mechanical damage and dehydration compared with lettuce or rocket (Kader, 2002; Medina et al., 2012). Nitrate assimilation is relatively efficient, although spinach can still accumulate substantial nitrate depending on its physiological state (Chen et al., 2004; Santamaria, 2006). Postharvest behavior reflects its higher respiration rate and susceptibility to yellowing (Kader, 2002; Medina et al., 2012).

Rocket leaves are thin, highly transpiring, and metabolically active, which predisposes the crop to rapid water loss and limited postharvest longevity (**Table 2**) (Nicola et al., 2010). *Eruca* (salad rocket) deteriorates especially quickly due to its faster growth, higher stomatal density, and pronounced nitrate accumulation during rapid leaf expansion (Nicola et al., 2010; Bell et al., 2016). *Diplotaxis* (wild rocket) shares similar structural traits but typically exhibits slightly thicker, more resilient leaves and a more efficient nitrate-assimilation capacity, resulting in somewhat slower decline (Nicola et al., 2010; Bell et al., 2016; Villani et al., 2023). In both species, the glucosinolate-rich profile and high respiration rate influence sensory quality and oxidative processes after harvest (Bell et al., 2020). These combined structural and metabolic features explain the markedly faster deterioration of rocket compared with lettuce and spinach, with *Eruca* (salad rocket) generally showing the shortest shelf life.

Taken together, the biological characteristics of lettuce, spinach, and rocket create distinct postharvest trajectories that reflect their anatomy, physiology, and metabolic composition. Nitrate content, where relevant, integrates into this broader biological context, helping explain interspecific differences in physiological behavior and postharvest performance.

## 3 Environmental factors during greenhouse cultivation

### 3.1 Air temperature

Air temperature is a primary environmental variable regulating growth, nitrate metabolism, pigment stability, and structural integrity in leafy vegetables cultivated in greenhouses (Bian et al., 2020; Gruda et al., 2025). Temperature affects both carbon and nitrogen metabolism, with NR activity and chlorophyll synthesis being particularly temperature-sensitive (Stitt and Krapp, 1999; Yamori et al., 2014). Because the optimal temperature range for quality formation is generally narrower than that required for growth, temperature deviations, even if moderate, can lead to substantial changes in nitrate accumulation, pigment concentration, and subsequent shelf-life performance (Gruda et al., 2025). Understanding species-specific temperature responses is therefore essential for designing climate regimes that safeguard both nutritional quality and postharvest longevity.

Lettuce grows optimally at moderate temperatures (15–22 °C), where nitrate assimilation proceeds efficiently and leaf tissue develops adequate firmness (**Table 3**) (He et al., 2010; Agüero et al., 2011a; Yamori et al., 2014). When temperatures exceed 24–26 °C, NR activity decreases and leaves tend to accumulate higher nitrate concentrations (He et al., 2010; Bian et al., 2020). Elevated night temperatures intensify respiration, weaken pigment stability, and accelerate the onset of browning and loss of turgor after harvest (Moreira et al., 2006; Manolopoulou et al., 2010; Agüero et al., 2011a). By contrast, temperatures below 12 °C slow metabolic processes and may lead to internal discoloration disorders such as pinking (Manolopoulou et al., 2010; Agüero et al., 2011a; Atkinson et al., 2013). A moderate positive day–night temperature difference (DIF) generally maintains pigment retention, firmness, and overall leaf structure, producing lettuce with improved storage potential (Manolopoulou et al., 2010; Agüero et al., 2011a; Atkinson et al., 2013).

**Table 3:** Effects of environmental factors during cultivation on nitrate accumulation, nutritional quality, and shelf life in greenhouse-grown lettuce.

Environmental factor	Main physiological effect(s)	Nitrate and nutritional quality	Shelf life/postharvest performance	Key references
Air T & DIF	Regulates leaf expansion, respiration, and NR activity	15–20 °C: balanced growth and NR; >22–24 °C: ↓ NR → ↑ NO_3_^−^; <12 °C: slower metabolism, risk of pigment imbalance	Warm nights: ↑ respiration → ↓ shelf life; positive DIF (warmer days, cooler nights): ↑ firmness, ↓ discoloration	(Dapoigny et al., 2000; Kader, 2002; Chen et al., 2004; Moreira et al., 2006; He et al., 2010; Manolopoulou et al., 2010; Agüero et al., 2011a; Yamori et al., 2014; Gent, 2016b; Ku et al., 2016)
Root-zone T	Controls root activity, N uptake, and water absorption	Optimal root-zone T (18–20 °C): steady N uptake; low root-zone T: ↓ uptake, possible ↓ NO_3_^−^ but also ↓ growth	Too low root-zone T: ↑ risk of tip burn–like symptoms and delayed growth; mild suboptimal T: compact plants with moderate storability	(Tan et al., 2002; He et al., 2010; Bumgarner et al., 2012; Yamori et al., 2014; Carotti et al., 2021; Levine et al., 2023; Zhao et al., 2025)
Light intensity, photoperiod & spectral quality	Drives photosynthesis, C supply, and NR induction	Sufficient DLI & B/R balance: ↑ NR → ↓ NO_3_^−^; low light: ↑ NO_3_^−^, ↓ phenolics, ↓ ascorbic acid; targeted B/UV-A: ↑ phenolics	Adequate light: ↑ pigments, ↑ antioxidant capacity, ↑ shelf life; low light: ↑ discoloration, ↓ visual quality	(Gaudreau et al., 1995; Zhou et al., 2009; Samuolienė et al., 2012; Gent, 2014; Mampholo et al., 2016; Son et al., 2017; Loi et al., 2020; Viršilė et al., 2020; Liang et al., 2022; Villani et al., 2023)
CO_2_ enrichment	Enhances photosynthetic C assimilation	Moderate CO_2_ (700–900 µmol mol^−1^): ↑ sugars, ↑ DM; improved C:N ratio may favor NO_3_^−^ reduction	↑ tissue density, ↑ firmness; with correct T and light: mild shelf-life extension via stronger cell walls	(Mortensen, 1987; Poorter and Nagel, 2000; Pérez-López et al., 2015; Chen et al., 2021)
RH, VPD & air movement	Influences transpiration, Ca transport, and cuticle formation	Moderate VPD (0.7–1.0 kPa): balanced hydration and nutrient distribution; very high RH: diluted tissues, potential ↑ NO_3_^−^ per FW	High RH, low air movement: soft leaves, ↑ decay; moderate air movement: ↑ cuticle integrity, ↑ shelf life	(Grange and Hand, 1987; Arita and Suslow, 2002; Santamaria, 2006; Amitrano et al., 2021, 2022, 2025)
Irrigation regime & water status	Governs leaf WC, osmotic balance, and succulence	Uniform supply: stable NO_3_^−^ per FW; mild deficit: ↑ DM, slight ↑ phenolics and ascorbic acid; severe deficit: ↓ growth, possible concentration of NO_3_^−^ per FW	Mildly reduced irrigation: ↑ firmness, better structure; severe deficit: wilting, ↓ marketability, shorter shelf life	(Caser, 2005; Oh et al., 2010; Luna et al., 2012; Malejane et al., 2018; Michelon et al., 2020; Abd–Elrahman et al., 2022; Lee et al., 2022)
Fertigation (N, K, Ca, EC, moderate salinity)	Sets N availability, K:Ca balance, and ionic strength	High N: ↑ NO_3_^−^; balanced N with moderate EC: ↓ NO_3_^−^ per FW, ↑ phenolics; adequate Ca & K: ↑ ascorbic acid, better nutrient balance	Adequate Ca: ↓ tip burn, ↑ structural integrity, ↑ shelf life; high EC: smaller but firmer heads; extreme EC: ↑ physiological stress & decay	(Barta and Tibbitts, 1991; Gent, 2003; White, 2003; Scuderi et al., 2009; Zörb et al., 2014; Sublett et al., 2018; Tabaglio et al., 2020; Conversa et al., 2021; Kappel et al., 2021; Weil et al., 2021; Beacham et al., 2023)
Canopy management (density, pruning, harvest stage)	Alters light interception, self-shading, and microclimate	Dense canopy: shaded inner leaves → ↑ NO_3_^−^, ↓ pigments; optimal density: uniform quality; harvest at slightly mature stage: better nutrient profile vs very young leaves	High density: ↑ RH → ↑ decay after harvest; optimal density and harvest stage: improved uniformity and shelf life	(Marcelis et al., 1998; Ryder, 1999; Kays and Paull, 2004; Cometti et al., 2011; Ilić et al., 2017; Ahmed et al., 2020; Bian et al., 2020; Jadhav et al., 2025)
Seasonality (T, light, RH)	Defines combined microclimate trajectory	Winter (low light, cool T): ↑ NO_3_^−^, ↑ firmness; Spring/autumn: balanced NO_3_^−^ and quality; summer (high T): risk of bolting, ↑ defects	Cool seasons: firmer leaves, sometimes longer shelf life; warm seasons: faster senescence, more tip burn and physiological disorders	(Dapoigny et al., 2000; Escobar-Gutiérrez et al., 2002; Kays and Paull, 2004; Beacham et al., 2023; Gruda et al., 2025)
Cover materials & shading	Modulate light diffusion, total radiation and heat load	Diffuse covers: ↑ light penetration, ↑ NR, ↓ NO_3_^−^; moderate shading in summer: protected pigments, maintained ascorbic acid	Diffuse light: ↑ color uniformity, ↑ shelf life; heavy shading: ↑ NO_3_^−^, ↓ structural strength, ↓ shelf life	(Kittas et al., 1999; Zhao and Carey, 2009; Ilić et al., 2017; Riga et al., 2019; Kim et al., 2022)
Biostimulants & mineral fortification (Ca, Si, etc.)	Enhance root function, antioxidant systems, and cell wall strength	Some biostimulants: ↑ phenolics, ↑ ascorbic acid; Ca/Si fortification: improved membrane stability, possible ↓ NO_3_^−^ per FW	↑ firmness, ↓ browning and decay, extended shelf life through stronger tissues and better oxidative status	(Campbell, 1999; Kader, 2002; White, 2003; Ma and Yamaji, 2006; Santamaria, 2006; White and Broadley, 2009; Rouphael et al., 2018; Mughunth et al., 2024; Atero-Calvo et al., 2025; Madjar et al., 2025)
Pest/disease pressure & residue-free IPM strategies	Induce defense pathways, affect assimilation	Mild biotic stress or elicitors: ↑ phenolics; severe infections: ↓ sugars and vitamins; NR can be suppressed under heavy stress	IPM and reduced pesticide use: ↓ decay risk (less wounding), ↑ consumer acceptability; severe outbreaks: strong shelf-life reduction	(Prokopová et al., 2010; Moreno-Escamilla et al., 2017; Collinge et al., 2022; Nietupski et al., 2022; Macioszek et al., 2023; Peng and Simko, 2023)
Major climate-factor interactions	Combined T × light × RH × CO_2_ effects on metabolism	Balanced T + adequate light + moderate N: ↓ NO_3_^−^, ↑ pigments & phenolics; adverse combinations (low light + high N + high RH): ↑ NO_3_^−^, ↓ nutritional density	Moderate integrated climate control: ↑ firmness and color; extreme combinations (heat + high RH) → rapid quality loss	(Miyagi et al., 2017; Bian et al., 2020; Cozzolino et al., 2020; Endo et al., 2022; Liang et al., 2022; Fanourakis et al., 2025a; Gruda et al., 2025)
Climate change implications	Trend towards higher T, greater variability	More frequent heat waves → ↑ NO_3_^−^, ↑ bolting risk, ↑ physiological stress	Elevated T extremes: shortened harvest windows, ↑ postharvest losses, need for improved cooling	(Tudela, 2017; Bian et al., 2020; Guffanti et al., 2022; Hao, 2022; Yang et al., 2022)
Cultivar differences & genetic adaptation	Genotype-specific growth and metabolic traits	Genotypes vary in baseline NO_3_^−^, NR activity, pigment density, and phenolic profiles	Cultivars differ in firmness, discoloration susceptibility, and storage potential; scope for breeding low-NO_3_^−^, high-shelf-life types	(Burns et al., 2011; Damerum et al., 2020; Martínez-Ispizua et al., 2022a; Peng and Simko, 2023; Pumnuan et al., 2024)

Ascorbic acid, vitamin C; B, blue (spectra); Ca, calcium; CO_2_, carbon dioxide; DIF, day–night temperature difference; DLI, daily light integral; EC, electrical conductivity; IPM, integrated pest management; K, potassium; N, nitrogen; NO_3_^−^, nitrate; NR, nitrate reductase; R, red (spectra); RH, relative air humidity; Si, silicon; T, temperature; UV, ultraviolet; VPD, vapor pressure deficit; WC, water content. Arrows indicate direction of projected change (↑ increase, ↓ decrease).

Spinach performs best at cooler temperatures (12–18 °C), where chlorophyll levels, folates, and ascorbic acid are well maintained (**Table 4**) (Hardin et al., 2011; Gent, 2016b; Mudau et al., 2019). Temperatures above 20 °C increase respiration rate, reduce pigment stability, and can decrease nitrate reduction capacity, resulting in elevated nitrate accumulation in rapidly growing leaves (Aydin and Nalbantoglu, 2011; Yamori et al., 2014; Gent, 2016b). Sustained high temperatures also promote faster postharvest yellowing and softening due to turgor loss and cell-wall weakening (Tan and Francis, 1962; Mudau et al., 2019). At the lower end of the optimal range, leaf robustness improves and chlorophyll breakdown slows down, but prolonged temperatures below 10–12 °C may restrict growth (Boese and Huner, 1990; Chadirin et al., 2011; Mudau et al., 2019). A well-defined day–night temperature rhythm supports tissue integrity and enhances shelf life by slowing metabolic decline (Boese and Huner, 1990; Yamori et al., 2005; Mudau et al., 2019).

**Table 4:** Effects of environmental factors during cultivation on nitrate accumulation, nutritional quality, and shelf life in greenhouse-grown spinach.

Environmental factor	Main physiological effect(s)	Nitrate and nutritional quality	Shelf life/postharvest performance	Key references
Air T & DIF	Controls leaf expansion, respiration, and pigment stability	12–18 °C: optimal NR and pigment formation; >20 °C: ↑ respiration, ↓ pigment stability, potential ↑ NO_3_^−^; <10–12 °C: slowed growth, possible ↑ chlorophyll retention	High T: faster yellowing and softening; cool nights: delayed senescence, improved shelf life	(Boese and Huner, 1990; Yamori et al., 2005, 2006; Aydin and Nalbantoglu, 2011; Chadirin et al., 2011; Gent, 2016b; Mudau et al., 2019)
Root-zone T	Modulates root growth, N and water uptake	Optimal root-zone T (~18 °C): efficient N uptake and reduction; low root-zone T: ↓ uptake, potentially ↓ NO_3_^−^ but also ↓ vigor	Mildly cool root-zone T: compact plants with good texture; very low T: risk of root stress, poor postharvest performance	(Breimer, 1982; Chadirin et al., 2011; Ito et al., 2015; Gent, 2016b; Edelenbos et al., 2017; Wang et al., 2022)
Light intensity, photoperiod & spectral quality	Drives photosynthesis, folate and pigment biosynthesis	Adequate DLI: ↑ NR, ↓ NO_3_^−^, ↑ chlorophyll, ↑ folates, ↑ ascorbic acid; low light: ↑ NO_3_^−^, ↓ antioxidant capacity	Sufficient light: ↑ color retention, ↑ structural integrity, longer shelf life; low light: rapid discoloration during storage	(Hodges et al., 2004; Proietti et al., 2004; Ohashi-Kaneko et al., 2007; Bekaert et al., 2008; Mampholo et al., 2016; Utasi et al., 2019; Miao et al., 2023; Martínez-Moreno et al., 2024)
CO_2_ enrichment	Enhances C fixation and DM accumulation	Moderate CO_2_: ↑ sugars, ↑ DM; improved C:N ratio may favor NO_3_^−^ assimilation and ↑ antioxidant levels	Denser tissues, ↑ firmness; with good T and light: modest shelf-life extension	(Holbrook et al., 1993; Kader, 2002; Proietti et al., 2004; Jain et al., 2007; Seo et al., 2017)
RH, VPD & air movement	Influences transpiration, leaf thickness, and cuticle	Moderate VPD (0.7–1.0 kPa): balanced WC, good mineral distribution; high RH: thinner leaves, potential dilution of nutrients	High RH with limited airflow: ↑ decay and soft rot; moderate airflow: ↓ disease, improved shelf life	(Iwabuchi et al., 1996; Arkebauer, 2000; Gutiérrez-Rodríguez et al., 2012; Hardin et al., 2013; Oliveira et al., 2016; Amitrano et al., 2019; Batziakas et al., 2020; Siddique et al., 2021)
Irrigation regime & water status	Determines leaf succulence and WC dynamics	Uniform water: stable nutrient levels; mild deficit: ↑ DM, possibly ↑ phenolics; strong deficit: ↓ growth, apparent ↑ NO_3_^−^ per FW by concentration	Mild deficit: ↑ firmness, slightly better texture; strong deficit: wilting, rapid quality loss postharvest	(Colla et al., 2018; Reyes et al., 2018; Nasarullah et al., 2022; Sun et al., 2023; Gruda et al., 2025)
Fertigation (N, K, Ca, EC, moderate salinity)	Controls availability of N and key minerals	High N: ↑ NO_3_^−^; adjusted N with moderate EC: ↓ NO_3_^−^ per FW, ↑ phenolics and ascorbic acid; adequate K and Ca: balanced nutritional profile	Sufficient Ca: stronger cell walls, delayed yellowing; high EC: smaller but firmer leaves; excessive EC: stress and shorter shelf life	(Abubaker et al., 2010; Oliveira et al., 2016; Xu and Mou, 2016; Deveci et al., 2019; Bian et al., 2020; Di Mola et al., 2020; Weil et al., 2021; Gülüt and Şentürk, 2024; Naik et al., 2025)
Canopy management (density, harvest stage)	Alters mutual shading and leaf age structure	High density: shaded lower leaves with ↑ NO_3_^−^ and ↓ pigments; optimal density: more uniform NO_3_^−^ and nutrient distribution	Overcrowding: ↑ RH → ↑ decay; optimal density and timely harvest: improved storability	(Cantliffe, 1972; Kays and Paull, 2004; Proietti et al., 2004, 2013; Utasi et al., 2019; Bian et al., 2020)
Seasonality (T, light, RH)	Shapes combined climate trajectory	Winter: low light → ↑ NO_3_^−^, but firm leaves; cool seasons: ↑ pigments but slower growth; warm seasons: quicker growth, ↓ pigments	Cool seasons: often longer shelf life; warm seasons: rapid yellowing and microbial spoilage	(Kays and Paull, 2004; Proietti et al., 2009; Mudau et al., 2019; Bian et al., 2020; Gruda et al., 2025; Gulyás et al., 2025)
Cover materials & shading	Modify total and diffuse light and heat load	Diffuse covers and moderate shading: ↑ light uniformity, stable pigments, potential ↓ NO_3_^−^; heavy shading: ↑ NO_3_^−^, ↓ folates and ascorbic acid	Diffuse light: ↑ color uniformity and firmness; excessive shading: softer, paler leaves, shorter shelf life	(Proietti et al., 2004; Mola et al., 2022; Miao et al., 2023; Semenova et al., 2023; Martínez-Moreno et al., 2024)
Biostimulants & mineral fortification	Stimulate root growth, antioxidant systems	Certain biostimulants: ↑ phenolics, ↑ ascorbic acid; Ca/Si: ↑ membrane stability, possible ↓ NO_3_^−^ fraction	↑ resistance to mechanical and oxidative damage, delayed yellowing, longer shelf life	(White, 2003; Ma and Yamaji, 2006; Fan et al., 2011; Du Jardin, 2015; Rouphael et al., 2018; Rouphael and Colla, 2020; Khosa et al., 2023)
Pest/disease pressure & residue-free IPM strategies	Trigger defense responses, alter resource allocation	Mild elicitation: ↑ phenolics; heavy disease: ↓ chlorophyll, ↓ vitamins, possible ↓ NO_3_^−^ via reduced uptake	Effective IPM: ↓ decay, improved visual quality; severe outbreaks: strong shelf-life reduction	(Schultz et al., 2013; Baenas et al., 2014; Fagard et al., 2014; Lu and Yao, 2018; Lawal et al., 2025)
Major climate-factor interactions	Combined effect of T × light × RH × N	High N + low light: ↑ NO_3_^−^, ↓ pigments; adequate light + moderate N + cool T: ↓ NO_3_^−^, ↑ folate and ascorbic acid	Balanced climate management: ↑ firmness and color; combined heat and high RH: fast softening and decay	(Proietti et al., 2004; Steele, 2004; Lester et al., 2010; Chang et al., 2013; Gent, 2016a; Han et al., 2023; Gulyás et al., 2025)
Climate change implications	More frequent heat episodes and extremes	Heat waves: risk of ↑ NO_3_^−^, ↓ pigment and vitamin retention	Elevated T extremes: shortened harvest and storage windows, ↑ postharvest losses	(Proietti et al., 2004; Medina et al., 2012; Mudau et al., 2015; Dong et al., 2018; Bian et al., 2020; Han et al., 2023)
Cultivar differences & genetic adaptation	Genotype-specific N metabolism and pigment capacity	Strong cultivar differences in NO_3_^−^ accumulation, folate, pigment levels, and oxalate content	Varied yellowing rates, tissue robustness, and microbial susceptibility; potential to select low-NO_3_^−^, high-shelf-life genotypes	(Yosefi et al., 2010; Koh et al., 2012; Solberg and Axelsson, 2015; Wang et al., 2018a; Weiss and Gruda, 2025)

Ascorbic acid, vitamin C; Ca, calcium; CO_2_, carbon dioxide; DIF, day–night temperature difference; DLI, daily light integral; EC, electrical conductivity; IPM, integrated pest management; K, potassium; N, nitrogen; NO_3_^−^, nitrate; NR, nitrate reductase; RH, relative air humidity; Si, silicon; T, temperature; VPD, vapor pressure deficit; WC, water content. Arrows indicate direction of projected change (↑ increase, ↓ decrease).

Rocket exhibits a broad but highly responsive temperature range, with *Eruca* (salad rocket) showing stronger nitrate fluctuations than *Diplotaxis* (wild rocket) (**Tables 5**, **6**) (Ferrante et al., 2003; Weightman et al., 2012; Caruso et al., 2018). Temperatures of 15–22 °C support balanced growth and adequate nitrate reduction in both species, sustaining high levels of glucosinolates and phenolics (Ferrante et al., 2003; Weightman et al., 2012; Ku et al., 2016). When temperatures exceed 24–25 °C, *Eruca* (salad rocket) rapidly accumulates very high nitrate due to accelerated growth and limited NR activity, whereas *Diplotaxis* (wild rocket) shows a more moderate increase (Ferrante et al., 2003; Podetta et al., 2011; Weightman et al., 2012). Elevated temperatures also intensify respiration and water loss, leading to fast wilting, especially in *Eruca* (salad rocket) (Ferrante et al., 2003; Frezza et al., 2010; Nicola et al., 2010). Cooler temperatures (10–15 °C) help preserve pigment stability and structural integrity in both species, though excessively low temperatures slow expansion and reduce uniformity, with *Eruca* (salad rocket) generally more affected than *Diplotaxis* (wild rocket) (Ferrante et al., 2003; Edelenbos et al., 2017).

**Table 5:** Effects of environmental factors during cultivation on nitrate accumulation, nutritional quality, and shelf life in greenhouse-grown *Eruca sativa* (salad rocket).

Environmental factor	Main physiological effect(s)	Nitrate and nutritional quality	Shelf life/postharvest performance	Key references
Air T & DIF	Controls rapid leaf expansion, respiration, sulfur metabolism	15–20 °C: balanced NO_3_^−^ and glucosinolates; >22–25 °C: very fast growth, ↓ NR → ↑ NO_3_^−^; <10–12 °C: slowed growth, sometimes ↑ pungency	High T: very rapid wilting; warm nights accelerate yellowing; positive DIF improves firmness	(Ferrante et al., 2003; Kim and Ishii, 2007; Frezza et al., 2010; Nicola et al., 2010; Doležalová et al., 2013; Urlić et al., 2017)
Root-zone T	Modulates root activity and N uptake	18–20 °C: strong N uptake → ↑ NO_3_^−^; slightly cooler root-zone T: moderated uptake → ↓ NO_3_^−^ per FW	Too low root-zone T: thin, fragile leaves; mild cooling: firmer leaves, slightly better shelf life	(Ferrante et al., 2003; Nicola et al., 2010; Urlić et al., 2017; He et al., 2022; Kim et al., 2025)
Light intensity, photoperiod & spectral quality	Drives photosynthesis and glucosinolate synthesis	Adequate DLI + B/UV-A: ↑ phenolics & glucosinolates, ↓ NO_3_^−^; low light: ↑ NO_3_^−^, ↑ succulence, ↓ pungency	Adequate light: higher firmness, color, antioxidant capacity; low light: pale, watery leaves with short shelf life	(Hodges et al., 2004; Jin et al., 2009; Pasini et al., 2011; Scharff et al., 2012; Cavaiuolo and Ferrante, 2014; Wagstaff, 2014; Mampholo et al., 2016; Bell et al., 2020; Elmardy et al., 2021)
CO_2_ enrichment	Enhances C assimilate supply	Moderate CO_2_ (700–900 µmol mol^−1^): ↑ DM, ↑ C:N ratio → improved NO_3_^−^ reduction	Slightly thicker leaves, slower water loss if T and VPD are well managed	(Stitt and Krapp, 1999; Kader, 2002; Dong et al., 2018; Schmidt and Zinkernagel, 2021)
RH, VPD & air movement	Regulates transpiration and leaf WC	Moderate VPD (~0.8–1.0 kPa): balanced WC; high RH: very succulent leaves → possible nutrient dilution	High RH: fragile tissues, poor shelf life; adequate airflow improves structural integrity	(Arkebauer, 2000; Kader, 2002; Tsirogiannis et al., 2013; Nikzad et al., 2023; Yu et al., 2023; Lee et al., 2024; Hutchinson et al., 2025; Zeinalipour et al., 2025)
Irrigation regime	Controls WC and osmotic balance	Uniform irrigation: steady NO_3_^−^; mild deficit: ↓ NO_3_^−^, ↑ phenolics & glucosinolates; strong deficit: stress-induced concentration of NO_3_^−^ per FW	Mild deficit improves firmness; severe deficit → fast wilting, strong loss of marketability	(Tsirogiannis et al., 2013; Guijarro-Real et al., 2019; Nikzad et al., 2023; Schiattone et al., 2023; Lee et al., 2024; Cakmakci, 2025)
Fertigation (N, K, Ca, EC)	Dictates N load and mineral balance	High N: ↑↑ NO_3_^−^; balanced N + moderate EC: ↓ NO_3_^−^, ↑ glucosinolates; Ca improves tissue integrity	Adequate Ca & EC: better structure; high N or EC: rapid yellowing and decay	(Costa et al., 2025; Omirou et al., 2012; Yang et al., 2021)
Canopy management	Influences shading and leaf age	High density: shaded, NO_3_^−^-rich leaves; optimal density + timely harvest: balanced NO_3_^−^	High-density canopy → high RH and faster decay; optimal density improves shelf life	(Marcelis et al., 1998; Nicola et al., 2005; Siomos and Koukounaras, 2007; Bantis et al., 2020)
Seasonality	Integrates T, light, RH variation	Winter: ↑ NO_3_^−^, ↓ glucosinolates; spring/autumn: balanced profile; summer: bolting risk, heat stress	Winter leaves firmer but more NO_3_^−^-rich; summer: very short shelf life	(Hall et al., 2013; Jakše et al., 2013; Cavaiuolo and Ferrante, 2014; Stajnko et al., 2022)
Cover materials & shading	Alter light diffusion and T	Diffuse covers: ↑ NR → ↓ NO_3_^−^; moderate shading protects pigments; heavy shading ↑ NO_3_^−^	Diffuse light: uniformity & slightly longer shelf life; heavy shade: soft leaves	(Santamaria et al., 2001; Hemming et al., 2008; Poorter et al., 2009; Bell and Wagstaff, 2014; Frary, 2015; Paradiso et al., 2023b)
Biostimulants & fortification	Stimulate roots & defense	Some elicitors: ↑ phenolics & glucosinolates, can ↓ NO_3_^−^; Ca/Si strengthen membranes	↑ firmness, ↓electrolyte leakage, slower yellowing	(White, 2003; Ma and Yamaji, 2006; Bell and Wagstaff, 2019; El-Nakhel et al., 2023; Franzoni et al., 2023; Tallarita et al., 2025)
Pest/disease pressure, IPM	Induces defenses	Mild elicitation: ↑ glucosinolates; severe stress: ↓ C compounds, may ↓ NO_3_^−^ via ↓ growth	Effective IPM reduces decay; severe outbreaks → near-immediate deterioration	(Bennett et al., 2006; Martínez-Sánchez et al., 2006; Schultz et al., 2013; Fagard et al., 2014; Lu and Yao, 2018; Lawal et al., 2025)
Major climate interactions	T × light × RH × N	Low light + high N: ↑↑ NO_3_^−^; adequate light + moderate N + moderate RH: ↓ NO_3_^−^, ↑ glucosinolates	Balanced climate improves firmness; extremes → extremely short shelf life	(Frezza et al., 2010; Omirou et al., 2012; Cavaiuolo and Ferrante, 2014; Bell et al., 2020; Bian et al., 2020; Signore et al., 2020; Stajnko et al., 2022)
Climate change implications	Higher means & extremes	Heat → ↑ NO_3_^−^, ↓ glucosinolates, ↑ bolting	Narrow safe harvest window under warming trends	(Cavaiuolo and Ferrante, 2014; Bell et al., 2020; Signore et al., 2020; Tripodi et al., 2024)
Cultivar differences	Genotype-specific profiles	Strong variation in NO_3_^−^ baseline, glucosinolate content, phenolics	Large differences in wilting, electrolyte leakage, decay; opportunities for improved cultivars	(Koukounaras et al., 2007; Siomos and Koukounaras, 2007; Pasini et al., 2012; Bell et al., 2015; Signore et al., 2020)

Ascorbic acid, vitamin C; B, blue (spectra); Ca, calcium; CO_2_, carbon dioxide; DIF, day–night temperature difference; DLI, daily light integral; EC, electrical conductivity; IPM, integrated pest management; K, potassium; N, nitrogen; NO_3_^−^, nitrate; NR, nitrate reductase; RH, relative air humidity; Si, silicon; T, temperature; UV, ultraviolet; VPD, vapor pressure deficit; WC, water content. Arrows indicate direction of projected change (↑ increase, ↓ decrease).

**Table 6:** Effects of environmental factors during cultivation on nitrate accumulation, nutritional quality, and shelf life in greenhouse-grown *Diplotaxis tenuifolia* (wild rocket).

Environmental factor	Main physiological effect(s)	Nitrate and nutritional quality	Shelf life/postharvest performance	Key references
Air T & DIF	Controls moderate growth rate and pigment stability	15–20 °C: balanced NO_3_^−^; >24–25 °C: mild ↓ NR → moderate ↑ NO_3_^−^ (less than *Eruca*); <10 °C: slow growth, firmer texture	More heat-tolerant than *Eruca*; moderate T improves shelf life; warm nights accelerate yellowing	(Podetta et al., 2011; Weightman et al., 2012; Mastrandrea et al., 2016; Edelenbos et al., 2017; Caruso et al., 2018; Petrini et al., 2023)
Root-zone T	Modulates root function and N uptake	Root-zone T 18–20 °C: steady N uptake; slight cooling: ↓ NO_3_^−^ without major growth penalty	Slightly more robust leaves at cooler root-zone T; too low T → reduced growth	(Nicoletti et al., 2007; Karnoutsos et al., 2021; He et al., 2022; Mola et al., 2022; Mainos et al., 2023; Sodini et al., 2025)
Light intensity & spectrum	Strong regulator of pigmentation & phenolic synthesis	Good DLI + B/UV-A: ↑ phenolics, ↑ glucosinolates, ↓ NO_3_^−^; low light: ↑ NO_3_^−^ but less dramatic than *Eruca*	Stronger yellowing resistance; high light improves firmness; low light: softer leaves but still better than *Eruca*	(Hodges et al., 2004; Jin et al., 2009; Pasini et al., 2011; Cavaiuolo and Ferrante, 2014; Wagstaff, 2014; Bell et al., 2020; Elmardy et al., 2021; Artés-Hernández et al., 2022)
CO_2_ enrichment	Enhances DM accumulation and pigment retention	Moderate CO_2_: improved C:N ratio, ↓ NO_3_^−^, ↑ phenolics	Slightly improved firmness and reduced electrolyte leakage	(Peisker et al., 1998; Stitt and Krapp, 1999; Poorter and Nagel, 2000; Kader, 2002; Taub et al., 2008; Dong et al., 2018)
RH, VPD & air flow	Determines WC and cuticle integrity	Moderate VPD: optimal; high RH: ↑ WC but less extreme than *Eruca*; slight dilution effects possible	Higher structural resilience → slower wilting than *Eruca*; good airflow delays decay	(Arkebauer, 2000; Kader, 2002; Tsirogiannis et al., 2013; Nikzad et al., 2023; Yu et al., 2023; Lee et al., 2024; Hutchinson et al., 2025; Zeinalipour et al., 2025).
Irrigation regime	Regulates succulence & DM	Mild deficit: ↑ phenolics, ↑ glucosinolates, ↓ NO_3_^−^; severe deficit stresses leaves	Mild deficit improves texture; severe deficit → wilting, leaf collapse	(Bonasia et al., 2017; Guijarro-Real et al., 2019; Schiattone et al., 2023; Tripodi et al., 2024)
Fertigation	Sets N load and mineral balance	High N: ↑ NO_3_^−^ but still lower than *Eruca*; balanced N + moderate EC: ↓ NO_3_^−^, ↑ glucosinolates & phenolics	Adequate Ca improves storability; very high EC or N → accelerated senescence	(Siomos and Koukounaras, 2007; Bonasia et al., 2017; Bell et al., 2020; Candido et al., 2023; Villani et al., 2023; Palumbo et al., 2024)
Canopy management	Influences shading and leaf uniformity	High density: ↑ NO_3_^−^ & ↓ pungency; lower density: better balance	Denser canopies still show better resilience than *Eruca*	(Marcelis et al., 1998; Weightman et al., 2012; Caruso et al., 2018; Iammarino et al., 2022; Khoramizadeh et al., 2024)
Seasonality	Light–T–RH interactions	Winter: ↑ NO_3_^−^ but less extreme; spring/autumn: high quality; summer: some heat sensitivity	Generally better shelf life than *Eruca* across seasons	(Hall et al., 2013; Cavaiuolo and Ferrante, 2014; Jasper et al., 2020; Gruda et al., 2025)
Cover materials & shading	Modify light diffusion	Diffuse covers: ↑ NR, ↓ NO_3_^−^, ↑ glucosinolates	Improved uniformity and shelf life; over-shading: softer leaves	(Pattanayak and Chatterjee, 1998; Hemming et al., 2008; Caruso et al., 2020; Signore et al., 2020; Mola et al., 2022; Paradiso et al., 2023a)
Biostimulants & fortification	Enhance antioxidant metabolism	Elicitors: ↑ phenolics, ↑ glucosinolates; Ca/Si: ↑ membrane stability	↑ firmness, ↓ electrolyte leakage, slower yellowing	(Kader, 2002; White, 2003; Rouphael et al., 2018; Romano et al., 2022; Candido et al., 2023; Di Mola et al., 2023; Tallarita et al., 2025)
Pest/disease pressure	Activates defenses	Mild elicitation: ↑ glucosinolates; severe stress: ↓ sugars & pigments	Slower deterioration than *Eruca*, but still sensitive	(Kader, 2002; Martínez-Sánchez et al., 2006; Schultz et al., 2013; Bell and Wagstaff, 2014; Wagstaff, 2014; Lu and Yao, 2018)
Major climate interactions	T × light × N × RH	Low light + high N: ↑ NO_3_^−^, but less extreme; adequate light + moderate N: ↓ NO_3_^−^ strongly	Good microclimate control supports significantly longer shelf life	(Cavaiuolo and Ferrante, 2014; Bian et al., 2020; Signore et al., 2020; Conversa et al., 2021; Paradiso et al., 2023a; Palumbo et al., 2024; Gruda et al., 2025)
Climate change implications	Warming trends	Heat stress: moderate ↑ NO_3_^−^, ↓ pigment stability	Shelf life shortened under prolonged heat	(Siomos and Koukounaras, 2007; Cavaiuolo and Ferrante, 2014; Bonasia et al., 2017; Signore et al., 2020; Alotaibi, 2025)
Cultivar differences	Genotypic variation	Large differences in NO_3_^−^, glucosinolates, phenolics; often more stable than *Eruca*	Strong variation in wilting and electrolyte leakage among cultivars	(Ferrante et al., 2003; Cavaiuolo and Ferrante, 2014; Bell et al., 2017a; Stajnko et al., 2022; Kurina et al., 2025)

Ascorbic acid, vitamin C; B, blue (spectra); Ca, calcium; CO_2_, carbon dioxide; DIF, day–night temperature difference; DLI, daily light integral; EC, electrical conductivity; IPM, integrated pest management; K, potassium; N, nitrogen; NO_3_^−^, nitrate; NR, nitrate reductase; RH, relative air humidity; Si, silicon; T, temperature; VPD, vapor pressure deficit; WC, water content. Arrows indicate direction of projected change (↑ increase, ↓ decrease).

Overall, temperature determines the balance between growth and the accumulation of quality-related metabolites in lettuce, spinach, and rocket. Warmer conditions promote biomass accumulation but risk increasing nitrate levels and reducing pigment and antioxidant retention, thereby compromising shelf life. Cooler temperatures enhance leaf firmness and metabolic resilience but slow overall biomass accumulation when they fall below species-specific thresholds. Effective thermal management in greenhouses therefore requires maintaining temperatures within a narrow quality-oriented range and ensuring sufficient day–night variation to support both nitrate reduction and postharvest performance.

### 3.2 Root-zone temperature

Root-zone temperature (RZT) is a critical yet often underestimated component of greenhouse climate management, directly influencing nutrient uptake, water absorption, root activity, and metabolic balance (Sakamoto and Suzuki, 2015; Yamori et al., 2022). Because nitrate assimilation depends on both uptake and reduction, root temperature plays a central role in determining nitrate accumulation in leafy vegetables (Sakamoto and Suzuki, 2015; Bian et al., 2020; Yamori et al., 2022). RZT also affects leaf turgor, tissue density, pigment retention, and susceptibility to postharvest deterioration (He et al., 2010; Sakamoto and Suzuki, 2015; Bian et al., 2020). Compared with aerial temperature, the root zone typically experiences smaller fluctuations but can diverge substantially due to slab temperature, substrate properties, irrigation temperature, and ground contact (Cross et al., 2025). Maintaining species-appropriate RZT is essential for ensuring uniform growth, moderated nitrate levels, and favorable shelf-life outcomes.

In lettuce, moderate RZTs between 18 and 20 °C promote steady nutrient uptake and efficient nitrate reduction (**Table 3**) (Bumgarner et al., 2012; Yamori et al., 2022). When the root zone cools below 14–15 °C, water absorption declines, nitrate uptake slows disproportionally, and growth becomes increasingly restricted, often leading to smaller but firmer heads (Tan et al., 2002; He et al., 2013; Levine et al., 2023). Extremely cool RZT may slightly reduce nitrate concentration but at the expense of pigment development and uniformity (Tan et al., 2002; Bumgarner et al., 2012; Levine et al., 2023). Conversely, warm RZT above 22–24 °C results in excessive water uptake, more succulent leaves, and a tendency toward higher nitrate accumulation due to reduced nitrate assimilation capacity (Tan et al., 2002; He et al., 2013; Carotti et al., 2021). These physiological imbalances can also translate into softer texture and increased susceptibility to postharvest browning and decay (Manolopoulou et al., 2010; Carotti et al., 2021).

Spinach, being a cool-adapted species, responds favorably to RZTs around 16–18 °C, which support strong root activity and balanced nitrate metabolism (**Table 4**) (Chadirin et al., 2011; Ito et al., 2015; Wang et al., 2022). Slightly cooler root zones (14–16 °C) maintain good tissue firmness and help preserve pigments and antioxidant levels (Chadirin et al., 2011; Ito et al., 2015; Wang et al., 2022). However, very low RZT (<12 °C) slows nutrient uptake, compromises root function, and often produces uneven leaf expansion (Ito et al., 2015; Gent, 2016a; Wang et al., 2022). On the warmer end, root temperatures above 20–22 °C accelerate nitrate uptake but not its reduction, leading to elevated nitrate accumulation in fast-growing leaves (Breimer, 1982; Chadirin et al., 2011; Wang et al., 2022). Warm roots also promote softer tissues and predispose spinach to rapid yellowing and microbial spoilage during storage (Chadirin et al., 2011; Mudau et al., 2019; Wang et al., 2022).

Rocket exhibits fast root metabolism and a high nitrogen-uptake capacity, which makes both species highly responsive to RZT, though *Eruca* (salad rocket) reacts more strongly (**Tables 5**, **6**) (Karnoutsos et al., 2021; He et al., 2022). Optimal RZT near 18–20 °C supports rapid growth, strong nitrate uptake, and adequate nitrate reduction in both taxa (Tsirogiannis et al., 2013; Kim et al., 2025). When the root zone becomes too warm (>22–24 °C), *Eruca* (salad rocket) shows a pronounced mismatch between uptake and NR activity, leading to very high nitrate accumulation and the development of water-rich, fragile tissues that wilt rapidly after harvest (Karnoutsos et al., 2021; He et al., 2022). *Diplotaxis* (wild rocket) displays similar trends but typically accumulates less nitrate and maintains slightly firmer leaves under the same conditions (Caruso et al., 2018; Karnoutsos et al., 2021). Mildly cooler RZT (14–16 °C) improves firmness, pigment retention, and shelf life in both species, although excessively cool roots can temporarily slow expansion and reduce uniformity, with *Eruca* (salad rocket) generally more affected than *Diplotaxis* (wild rocket) (Karnoutsos et al., 2021; He et al., 2022).

In all three crops, RZT strongly influences nitrate accumulation and postharvest behavior by shaping the balance between nutrient uptake and metabolic utilization. Warm RZT favors rapid biomass production but increases the risk of high nitrate concentration and softer, more perishable leaves. Cooler RZT enhances tissue quality and storability but reduces growth when temperatures fall too low. Achieving optimal greenhouse performance therefore requires careful regulation of RZT through substrate selection, irrigation temperature, heating strategies, and cultivation system design.

### 3.3 Light intensity, photoperiod, and spectral quality

Light quantity and quality represent central determinants of carbon assimilation, nitrate metabolism, pigment biosynthesis, and the broader nutritional profile of leafy vegetables (Ohashi-Kaneko et al., 2007; Zhang et al., 2020). The daily light integral (DLI) governs the balance between nitrate uptake and reduction, since NR activity is tightly coupled to photosynthetic energy supply (Bian et al., 2020; Mayorga-Gomez et al., 2024; Rosli et al., 2025). Photoperiod influences developmental rate and leaf expansion, whereas spectral quality modulates physiological processes such as chlorophyll retention, phenolic synthesis, and the accumulation of species-specific secondary metabolites (Franklin and Quail, 2010; Dardick and Callahan, 2014; Bian et al., 2020). In greenhouse systems, where natural light is seasonally limiting, the interaction between light availability and nitrogen metabolism becomes a primary driver of both nitrate accumulation and postharvest performance (Albright et al., 2000; Lillo, 2004; Santamaria, 2006).

In lettuce, insufficient light, particularly under winter greenhouse conditions, results in reduced photosynthetic carbon supply and lower NR activity, leading to elevated nitrate accumulation in the leaves (**Table 3**) (Gaudreau et al., 1995; Gent, 2014; Bian et al., 2020; Viršilė et al., 2020). Low DLI also diminishes chlorophyll and phenolic concentrations, producing paler leaves with reduced antioxidant capacity and shorter shelf life (Zhou et al., 2009; Mampholo et al., 2016; Son et al., 2017; Loi et al., 2020). Conversely, higher light availability promotes stronger pigment formation, lower nitrate concentration, and firmer tissue structure (Zhou et al., 2009; Gent, 2014; Viršilė et al., 2020; Gude et al., 2021). Blue (B)-enriched spectra stimulate phenolic and flavonoid synthesis, improving nutritional value, while red (R) light supports efficient biomass accumulation (Zhou et al., 2009; Son and Oh, 2013; Loi et al., 2020). Excessively short photoperiods slow growth and increase nitrate, whereas moderate photoperiod extension (e.g., 14–16 hours with supplemental LEDs) improves nitrate reduction and color uniformity (Gaudreau et al., 1995; Zhou et al., 2009; Son and Oh, 2013; Viršilė et al., 2020).

Spinach exhibits a strong response to both DLI and spectral composition due to its high pigment content and rapid nitrogen turnover (**Table 4**) (Proietti et al., 2004; Utasi et al., 2019; Miao et al., 2023; Martínez-Moreno et al., 2024). Adequate light markedly enhances chlorophyll stability, folate concentration, and ascorbic acid levels while simultaneously lowering nitrate through improved nitrate assimilation (Proietti et al., 2004; Utasi et al., 2019). Under low-light conditions, spinach tends to accumulate high nitrate and displays accelerated yellowing after harvest due to weaker antioxidant protection (Hodges et al., 2004; Proietti et al., 2004; Mampholo et al., 2016; Utasi et al., 2019; Miao et al., 2023). B and ultraviolet (UV)-A wavelengths promote phenolic biosynthesis, contributing to improved oxidative protection and delayed senescence (Hodges et al., 2004; Mampholo et al., 2016; Utasi et al., 2019; Zhan et al., 2020; Martínez-Moreno et al., 2024). Photoperiod extension can increase total biomass and support more complete nitrate reduction, provided temperatures remain favorable (Steingröver et al., 1986; Proietti et al., 2004; Han et al., 2023).

Rocket performs best under moderate to high light availability, which supports efficient nitrate reduction, strong pigmentation, and robust synthesis of glucosinolates and phenolics (**Tables 5**, **6**) (Jin et al., 2009; Cavaiuolo and Ferrante, 2014; Elmardy et al., 2021). Under low DLI, nitrate accumulates rapidly and both pungency and antioxidant activity decline, with *Eruca* (salad rocket) typically showing a stronger increase in nitrate than *Diplotaxis* (wild rocket) (Jin et al., 2009; Pasini et al., 2011; Cavaiuolo and Ferrante, 2014; Elmardy et al., 2021). Spectral quality plays a key role: B and UV-A light markedly enhance glucosinolate and phenolic pathways in both species, improving nutritional density and characteristic flavor (Jin et al., 2009; Pasini et al., 2011; Cavaiuolo and Ferrante, 2014; Artés-Hernández et al., 2022). R light promotes leaf expansion but, when predominant, can dilute pigments and reduce bioactive concentrations (Jin et al., 2009; Cavaiuolo and Ferrante, 2014; Elmardy et al., 2021; Artés-Hernández et al., 2022). Photoperiod extension increases biomass but cannot compensate for low light intensity, often resulting in weaker flavor and reduced postharvest stability, especially in *Eruca* (salad rocket) (Hodges et al., 2004; Pasini et al., 2011; Elmardy et al., 2021). Overall, adequate light intensity combined with a balanced B/UV-A component is essential for maintaining low nitrate, high pungency, and good shelf-life performance in both rocket types (Cavaiuolo and Ferrante, 2014; Elmardy et al., 2021; Artés-Hernández et al., 2022).

Overall, the quantity and quality of light during greenhouse cultivation exert profound effects on nitrate accumulation, nutritional quality, and postharvest longevity in lettuce, spinach, and rocket. Higher DLI and balanced spectral composition, particularly with targeted B or UV-A supplementation, enhance pigment density, phenolic content, and nitrate reduction efficiency, leading to leaves with stronger structural integrity and longer shelf life. Conversely, low-light environments promote softer tissues, higher nitrate concentrations, and faster deterioration after harvest. Effective light management in greenhouses therefore requires integrating intensity, photoperiod, and spectral composition to achieve species-specific quality targets.

### 3.4 CO_2_ enrichment

CO_2_ enrichment is a fundamental greenhouse practice used to enhance photosynthetic carbon assimilation, dry matter (DM) accumulation, and overall productivity (Mortensen, 1987; Long et al., 2004). Because nitrate reduction is tightly linked to carbohydrate availability, elevated CO_2_ can influence nitrate metabolism indirectly by improving the carbon-to-nitrogen ratio in the plant (Stitt and Krapp, 1999; Foyer et al., 2001; Bloom et al., 2010). The interplay between CO_2_ enrichment, light availability, and temperature determines whether the additional carbon is effectively used to strengthen tissue structure, promote pigment formation, and reduce nitrate, or whether it simply accelerates growth without improving quality (Stitt and Krapp, 1999; Poorter and Nagel, 2000; Long et al., 2004). The effects of CO_2_ enrichment are therefore highly species-specific and strongly dependent on concurrent microclimatic conditions.

In lettuce, moderate CO_2_ enrichment (typically 600–900 µmol mol^−1^) increases leaf thickness, enhances sugar accumulation, and can support more efficient nitrate reduction when light conditions are adequate (**Table 3**) (Mortensen, 1987; Stitt and Krapp, 1999; Poorter and Nagel, 2000; Pérez-López et al., 2015). The resulting higher carbohydrate availability tends to improve pigment retention, increase firmness, and reduce susceptibility to postharvest browning (Pérez-López et al., 2015; Holley et al., 2022). However, under low light, elevated CO_2_ may accelerate growth without enhancing nitrate assimilation, leading to only modest changes, or even slight increases, in nitrate concentration (Pérez-López et al., 2015; Chen et al., 2021). Excessive CO_2_ enrichment in combination with warm temperatures may produce overly succulent leaves with shorter shelf life (Mortensen, 1987; Kader, 2002).

Spinach responds to CO_2_ enrichment with increased DM content, improved pigment density, and enhanced antioxidant status, provided that light is sufficient (**Table 4**) (Jain et al., 2007; Proietti et al., 2013; Seo et al., 2017). The higher carbon availability supports folate and ascorbic acid synthesis and may improve nitrate reduction indirectly (Stitt and Krapp, 1999; Seo et al., 2017; Dong et al., 2018). When CO_2_ enrichment coincides with adequate DLI, spinach leaves become structurally more robust and exhibit delayed yellowing postharvest (Kader, 2002; Proietti et al., 2013). Under low light, however, the benefits are reduced, and nitrate levels may not decline appreciably because NR activity remains light-limited despite the additional carbon supply (Stitt and Krapp, 1999; Proietti et al., 2013).

Rocket shows rapid biomass expansion under elevated CO_2_, with *Eruca* (salad rocket) generally responding more strongly than *Diplotaxis* (wild rocket) (**Tables 5**, **6**) (Dong et al., 2018; Schmidt and Zinkernagel, 2021). When CO_2_ enrichment is combined with adequate light, the increased carbon availability can support higher glucosinolate and phenolic synthesis and may slightly improve nitrate reduction through an improved carbon-to-nitrogen balance (Stitt and Krapp, 1999; Dong et al., 2018; Neugart et al., 2020). However, because both rocket species possess inherently high nitrate-uptake capacity, CO_2_ enrichment alone seldom decreases nitrate unless nitrogen supply and light intensity are simultaneously optimized (Stitt and Krapp, 1999; Santamaria, 2006; Schmidt and Zinkernagel, 2021). Elevated CO_2_ often increases leaf succulence, particularly in *Eruca* (salad rocket), which can weaken structural integrity and accelerate postharvest water loss unless accompanied by appropriate VPD and light management (Mortensen, 1987; Kader, 2002; Schmidt and Zinkernagel, 2021). Overall, CO_2_ enrichment enhances biomass and can improve phytochemical content under well-balanced conditions, but its effect on nitrate is conditional and often modest (Stitt and Krapp, 1999; Dong et al., 2018).

Overall, CO_2_ enrichment enhances photosynthetic performance and biomass productivity in all three leafy vegetables, but its effects on nitrate and quality depend critically on light and temperature. When integrated into a balanced greenhouse climate strategy, elevated CO_2_ can support improved pigment formation, structural integrity, and postharvest behavior. In contrast, CO_2_ enrichment under limiting light or suboptimal nutrient regimes may enhance growth without reducing nitrate or extending shelf life. Effective use of CO_2_ therefore requires careful coordination with other environmental parameters to deliver consistent quality improvements.

### 3.5 Relative air humidity, vapor pressure deficit, and air movement

RH quantifies the relative moisture content of the surrounding air, while the interrelated measure VPD (i.e., the difference between saturated leaf vapor pressure and ambient vapor pressure) integrates temperature effects and thus captures the actual driving force for water loss. RH, VPD, and air movement jointly regulate transpiration, leaf water balance, nutrient distribution, and cuticle development in greenhouse-grown leafy vegetables (Amitrano et al., 2021; Inoue et al., 2021; Yu et al., 2023). Because nitrate distribution depends on both xylem transport and metabolic reduction, these microclimatic factors significantly influence nitrate concentration as well as pigment retention, phenolic content, and tissue firmness (Chen et al., 2004; Santamaria, 2006; Amitrano et al., 2021, 2022). High RH reduces transpiration and calcium transport, producing softer tissues with higher WC, while excessively high VPD leads to dehydrating stress and impaired nutrient uptake (Amitrano et al., 2021, 2022). Air movement mitigates boundary-layer resistance, stabilizes leaf temperature, and reduces localized RH pockets that predispose leaves to decay after harvest (Arita and Suslow, 2002; Boulard, 2006; Katsoulas et al., 2007). The combined effects of RH, VPD, and airflow therefore exert strong, species-specific influences on both nutritional quality and shelf life.

Lettuce is highly sensitive to RH and VPD due to its thin cuticle and high surface-area-to-volume ratio (**Table 3**) (Grange and Hand, 1987; Amitrano et al., 2022, 2025). High RH combined with low air movement reduces transpiration, leading to diluted mineral concentrations, softer leaves, and an increased tendency for nitrate accumulation because nitrate reduction slows under low nitrate assimilation demand (Grange and Hand, 1987; Amitrano et al., 2022, 2025). These leaves often display reduced pigmentation and lower phenolic content, translating into shorter shelf life and greater susceptibility to browning and decay (Arita and Suslow, 2002; Amitrano et al., 2021). Moderate VPD (0.7–1.0 kPa) supports balanced hydration, firmer texture, and stable pigment development (Amitrano et al., 2021; Inoue et al., 2021). Airflow is critical for RH control within dense lettuce canopies, improving uniformity and reducing postharvest deterioration (Boulard, 2006; Katsoulas et al., 2007).

Spinach tolerates a somewhat broader RH range but is still strongly influenced by RH-driven changes in WC and tissue structure (**Table 4**) (Iwabuchi et al., 1996; Medina et al., 2012). High RH produces very turgid, water-rich leaves that are more prone to mechanical damage and microbial spoilage after harvest (Medina et al., 2012). Because spinach maintains relatively high NR activity, nitrate responses to RH are less extreme than in lettuce, yet prolonged low VPD conditions can still result in higher nitrate concentrations and reduced antioxidant content (Iwabuchi et al., 1996; Santamaria, 2006; Oliveira et al., 2016). Adequate airflow improves pigment stability, reduces local condensation risk, and slows yellowing by preventing microclimatic saturation around the leaf surface (Oliveira et al., 2016; Batziakas et al., 2020). Slightly elevated VPD enhances firmness without inducing excessive water loss (Iwabuchi et al., 1996; Oliveira et al., 2016).

Rocket is highly sensitive to RH and VPD due to its thin leaves, high stomatal density, and inherently fast transpiration dynamics (**Tables 5**, **6**) (Siomos and Koukounaras, 2007; Frary, 2015; Sinha et al., 2018; Lee et al., 2024). High RH produces very succulent, water-rich tissues that lack structural rigidity, making both *Eruca* (salad rocket) and *Diplotaxis* (wild rocket) highly prone to rapid wilting, electrolyte leakage (due to compromised membrane integrity), and microbial decay after harvest (Siomos and Koukounaras, 2007; Frezza et al., 2010; Kyriacou and Rouphael, 2018). Under low VPD, nitrate reduction is strongly inhibited, resulting in marked nitrate accumulation, while the synthesis of glucosinolates and phenolics often declines (Amitrano et al., 2021; Ampim et al., 2022; Gruda et al., 2025). In contrast, moderate VPD (≈0.8–1.0 kPa) improves leaf texture, promotes balanced hydration, enhances pigment and flavor-related secondary metabolites, and supports better postharvest performance (Siomos and Koukounaras, 2007; Bell et al., 2020; Amitrano et al., 2021). Airflow is especially critical in rocket canopies, as even brief localized RH spikes trigger water-soaked tissues and accelerate postharvest deterioration (Siomos and Koukounaras, 2007; Gómez et al., 2023). Overall, maintaining moderate VPD with sufficient air movement is essential to preserve structure, limit nitrate buildup, and enhance both nutritional and sensory quality in rocket (Siomos and Koukounaras, 2007; Frezza et al., 2010). These microclimate requirements must also be balanced against disease-suppression constraints, since climate regimes that optimize physiological quality may not always minimize pathogen pressure (Fanourakis et al., 2026). Species-specific trade-offs between climate optimization and disease control have been emphasized in recent analyses (Fanourakis et al., 2025b).

Overall, RH, VPD, and air movement exert pronounced effects on nitrate accumulation, nutritional quality, and postharvest longevity in lettuce, spinach, and rocket. Moderate VPD and adequate airflow generally promote firmer tissue, better pigment and phenolic profiles, and more effective nitrate reduction, while high RH with poor ventilation encourages soft, nitrate-rich tissues that deteriorate rapidly after harvest. Effective greenhouse climate management must therefore integrate RH control and airflow with temperature and light strategies to optimize quality outcomes in leafy vegetables.

### 3.6 Irrigation regime and water status

Irrigation management determines plant water status, leaf succulence, nutrient transport, and the structural properties of leafy vegetables during greenhouse cultivation (Locascio, 2005; Kim et al., 2016). Because nitrate is transported primarily through the transpiration stream and diluted by leaf WC, irrigation practices directly influence both nitrate concentration and broader nutritional attributes such as pigment density, phenolic content, and vitamin levels (Nyathi et al., 2018; He, 2024). Mild water deficits can promote higher DM content and improved tissue firmness, whereas over-irrigation generally increases WC, reduces structural integrity, and dilutes nutrient concentrations (Nyathi et al., 2018; He, 2024). Species differ in their sensitivity to deviations from optimal water availability, leading to distinct effects on nitrate accumulation, nutritional quality, and postharvest performance.

In lettuce, uniform irrigation supports consistent growth, balanced nitrate distribution, and stable pigment formation (**Table 3**) (Abd–Elrahman et al., 2022; Ruangsangaram et al., 2025; Silva et al., 2025). Excess irrigation produces highly succulent tissues with lower DM content, diluted mineral concentrations, and often higher nitrate per unit fresh weight due to reduced nitrate assimilation relative to uptake (Abd–Elrahman et al., 2022; Lee et al., 2022). Mildly reduced irrigation can increase firmness and sometimes enhance phenolic content, but substantial deficits cause wilting, smaller head formation, and increased variability in quality (Oh et al., 2010; Luna et al., 2012; Malejane et al., 2018). The narrow optimal hydration window makes lettuce particularly sensitive to water mismanagement during cultivation.

Spinach is capable of sustaining moderate water-deficit conditions better than lettuce due to its thicker leaves and higher baseline DM content (**Table 4**) (Kunicki et al., 2010; Gutiérrez-Rodríguez et al., 2012). Mild water limitation can increase pigment concentration, strengthen leaf texture, and slightly elevate antioxidant compounds (Zargar et al., 2023; Karić et al., 2024). However, severe deficit reduces leaf expansion and may lead to apparent increases in nitrate concentration on a fresh-weight basis due to reduced WC rather than actual accumulation (Bian et al., 2020; Seymen, 2021; Karić et al., 2024). Over-irrigated spinach becomes excessively turgid and mechanically fragile, predisposing it to rapid yellowing and microbial spoilage after harvest (Kuslu et al., 2016; Varner et al., 2024). Maintaining stable soil or substrate moisture supports both nutritional quality and postharvest durability (Gil, 2016; Sun et al., 2023; Gruda et al., 2025).

Rocket shows an exceptionally rapid physiological response to irrigation regime due to its high transpiration rate, thin leaves, and fast growth (**Tables 5**, **6**) (Tsirogiannis et al., 2013; Lee et al., 2024). Over-irrigation produces very succulent, water-rich tissues with weakened structure, reduced pungency, and diluted concentrations of glucosinolates and phenolics; nitrate levels often rise because nitrate uptake outpaces assimilatory capacity (Cavaiuolo and Ferrante, 2014; Schiattone et al., 2023; Lee et al., 2024). Mild water limitation can increase DM content, concentrate flavor compounds, and modestly enhance antioxidant levels, but the threshold between beneficial and harmful stress is narrow (Schiattone et al., 2023; Lee et al., 2024). Once water deficit becomes moderate to severe, rocket wilts rapidly, growth declines sharply, and postharvest deterioration accelerates (Nikzad et al., 2023; Lee et al., 2024). Given its extremely short shelf life, irrigation strategies must prevent excessive succulence while avoiding even brief periods of stress that produce fragile, electrolyte leakage-prone tissues (Siomos and Koukounaras, 2007; Frezza et al., 2010). Maintaining stable, moderate water availability is therefore essential to support both quality and storability in rocket.

Overall, irrigation regime and plant water status exert strong and immediate effects on nitrate concentration, nutritional composition, and postharvest behavior in greenhouse-grown leafy vegetables. Over-irrigation generally promotes higher nitrate levels, lower DM content, and weaker tissue structure, while moderate water limitation can improve firmness and certain nutritional attributes, provided it stays within species-specific tolerance limits. Effective irrigation management must balance growth, nutrient uptake and nitrate assimilation, and tissue quality to optimize both pre- and postharvest performance in lettuce, spinach, and rocket.

### 3.7 Fertigation: nitrogen, potassium, calcium, electrical conductivity, and moderate salinity

Fertigation management, encompassing nitrogen form and supply, potassium and calcium availability, and the overall electrical conductivity (EC) of the nutrient solution, plays a central role in nitrate accumulation, mineral composition, pigment formation, and tissue structure in leafy vegetables (Gent, 2003; Conversa et al., 2021; Corrado et al., 2021; Weil et al., 2021; Spyrou et al., 2025). Nitrogen directly governs nitrate uptake and reduction, while potassium influences osmotic balance, pigment stability, and ascorbic acid content (Zörb et al., 2014; Bian et al., 2020). Calcium is essential for membrane stability, cell-wall integrity, and postharvest firmness (White, 2003). Moderate elevations in EC can enhance DM and nutritional density, whereas excessive salinity suppresses growth and increases physiological stress (Conversa et al., 2021; Corrado et al., 2021). Species-specific physiological traits and nutrient-use efficiencies result in distinct responses of lettuce, spinach, and rocket to fertigation regimes.

Lettuce shows a strong, direct response to nitrogen supply, with high nitrate fertilization consistently producing elevated nitrate accumulation in leaves (**Table 3**) (Barros Júnior et al., 2020). Balanced nitrogen provision combined with moderate EC levels (e.g., 2.0–2.5 dS m^−1^) promotes higher DM content, improved pigment retention, and lower nitrate per unit fresh weight (Sublett et al., 2018; Conversa et al., 2021; Spyrou et al., 2025). Adequate potassium supply enhances color intensity and contributes to a more favorable sugar–acid balance, while sufficient calcium reduces susceptibility to tip burn and strengthens tissue firmness (Barta and Tibbitts, 1991; Barickman et al., 2016). Excessive EC or unbalanced nutrient ratios can impair water uptake, suppress leaf expansion, and predispose tissues to postharvest browning and rapid quality loss (Adhikari et al., 2019; Kappel et al., 2021).

Spinach efficiently assimilates nitrate under moderate supply but still displays increased nitrate accumulation when fertilization exceeds metabolic demand or when assimilation is limited by other factors such as light (**Table 4**) (Cantliffe, 1972; Bian et al., 2020). Balanced nitrogen and potassium fertilization promotes chlorophyll synthesis, folate formation, and ascorbic acid retention (Nemadodzi et al., 2017; Islam et al., 2025). Adequate calcium improves structural integrity and delays postharvest yellowing (Oliveira et al., 2016). Moderate EC levels support higher pigment and DM content, whereas excessive EC reduces leaf expansion and may elevate oxidative stress (Xu and Mou, 2016; Ferreira et al., 2018). Spinach generally benefits from precise fertigation control, as both deficiency and excess can impair nutritional quality and shorten shelf life (Gülüt and Şentürk, 2024).

Rocket exhibits very strong and immediate responses to fertigation because of its rapid nitrogen uptake and fast leaf expansion (**Tables 5**, **6**) (Yang et al., 2021; Palumbo et al., 2024). High nitrogen supply leads to pronounced nitrate accumulation, often the highest among the three leafy species, especially when light or VPD conditions limit nitrate reduction (Bian et al., 2020; Di Mola et al., 2020). Moderating nitrogen availability or using mixed nitrate–ammonium nutrition can reduce nitrate concentration while maintaining acceptable growth (Santamaria et al., 2001; Signore et al., 2020; Spyrou et al., 2025). Potassium plays a central role in supporting glucosinolate synthesis, pigment formation, and overall flavor intensity, whereas calcium strengthens membranes and reduces electrolyte leakage, wilting, and mechanical fragility after harvest, critical traits for this highly perishable crop (Thor, 2019; Sardans and Peñuelas, 2021). Moderate EC levels can enhance pungency, antioxidant compounds, and structural firmness, but excessive salinity quickly induces stress, reduces biomass accumulation, and results in softer, more deterioration-prone leaves (Pasini et al., 2011; Bonasia et al., 2017; Bell et al., 2020; Gioia et al., 2020). Effective fertigation management in rocket therefore requires balancing nitrogen supply with light and VPD conditions, maintaining adequate potassium and calcium, and avoiding high EC levels that compromise both yield and shelf life.

Overall, fertigation management is one of the most powerful tools for modulating nitrate concentration, nutritional composition, and postharvest performance in greenhouse-grown lettuce, spinach, and rocket. While nitrogen supply remains the primary driver of nitrate accumulation, coordinated adjustments of potassium, calcium, and EC improve structural integrity, pigment formation, and antioxidant capacity. Optimal fertigation strategies must therefore balance nutrient availability with the metabolic capacity of each species, to produce leafy vegetables of high nutritional value and extended shelf life.

### 3.8 Canopy management: density, spacing, leaf layering, and harvest stage

Canopy management in leafy vegetables encompasses plant density, spatial arrangement, leaf layering, and the timing of harvest (Marcelis et al., 1998; Ryder, 1999; Larcher, 2003). These factors shape light interception, self-shading patterns, microclimate formation within the canopy, and the physiological age of harvested tissue (Marcelis et al., 1998; Ryder, 1999; Larcher, 2003). Because nitrate reduction is highly light-dependent, canopy structure directly influences nitrate levels through its effects on photosynthetic carbon availability (Marschner, 2012; Frary, 2015). Dense canopies also elevate RH around leaf surfaces, altering pigment stability, phenolic synthesis, and tissue firmness (Kays and Paull, 2004; Monteith and Unsworth, 2013; Frary, 2015). Harvest stage determines the balance between young, nitrate-rich leaves and older, more structurally stable leaves (Ryder, 1999; Marschner, 2012; Frary, 2015). Together, these variables strongly affect both nutritional quality and postharvest performance.

In lettuce, plant density and leaf layering have marked effects on nitrate accumulation and pigment distribution (**Table 3**) (Ryder, 1999; Cometti et al., 2011). High-density planting increases shading in the lower canopy, resulting in elevated nitrate levels and reduced chlorophyll and phenolic concentrations in inner leaves (Kosma et al., 2013; Ilić et al., 2017). Excessive density also promotes higher RH around leaf surfaces, softening the tissue and increasing susceptibility to postharvest browning and decay (Ahmed et al., 2020; Jadhav et al., 2025). Optimal spacing improves light penetration, reduces nitrate concentration, and enhances color uniformity and firmness (Bian et al., 2020; Mutombo Arcel et al., 2023). Harvesting at the appropriate maturity stage ensures a balance between nutritional density and structural integrity, while overly early harvests yield nitrate-rich, fragile leaves (Ryder, 1999; Bian et al., 2020).

In spinach, which develops rosettes with relatively horizontal leaves, canopy density primarily affects light distribution and leaf physiological age (**Table 4**) (Proietti et al., 2004; Utasi et al., 2019; Bantis et al., 2020). Dense canopies promote nitrate accumulation in inner leaves and accelerate yellowing during storage due to limited light exposure and lower antioxidant content (Cantliffe, 1972; Yosefi et al., 2010). Moderate spacing improves pigment retention and promotes uniform leaf expansion (Proietti et al., 2004, 2013; Utasi et al., 2019). Harvest timing is particularly important: younger leaves tend to have higher nitrate and lower DM content, whereas slightly more mature leaves show improved structural integrity and delayed senescence after harvest (Cantliffe, 1972; Kays and Paull, 2004; Proietti et al., 2004, 2013). Overcrowding increases RH within the canopy and can lead to faster microbial deterioration during storage (Kays and Paull, 2004; Ahmed et al., 2020).

Rocket is highly sensitive to canopy structure because its thin leaves, fast growth, and highly active secondary-metabolite pathways respond sharply to light availability and localized RH (**Tables 5**, **6**) (Kays and Paull, 2004; Signore et al., 2020; Guffanti et al., 2022). High planting density reduces light penetration, especially to inner and lower leaves, leading to markedly elevated nitrate accumulation and reduced glucosinolate and phenolic synthesis. This results in milder flavor, lower antioxidant activity, and diminished nutritional value (Cavaiuolo and Ferrante, 2014; Caruso et al., 2018; Signore et al., 2020). Dense canopies also trap moisture, creating high RH microzones that accelerate postharvest wilting, electrolyte leakage, and microbial spoilage (Kays and Paull, 2004; Guffanti et al., 2022). Optimized spacing improves light distribution, enhances pungency and phenolic content, and promotes firmer, more resilient leaf structure (Marcelis et al., 1998; Colonna et al., 2016). Harvest stage is equally important: very young leaves tend to be softer, richer in water and nitrate, and more prone to rapid deterioration, whereas slightly more mature leaves show higher glucosinolate levels, stronger pigmentation, and better shelf-life performance (Kays and Paull, 2004; Caruso et al., 2018; Signore et al., 2020; Guffanti et al., 2022).

Overall, canopy management is a key determinant of nitrate concentrations, nutritional composition, and physiological resilience in lettuce, spinach, and rocket. Excessive density or suboptimal harvest timing promotes nitrate-rich, fragile tissues prone to rapid postharvest decline, whereas well-managed canopy structure ensures better light penetration, improved pigment and phenolic profiles, and greater firmness. Aligning plant spacing and harvest stage with species-specific growth patterns is therefore essential for achieving consistent quality and extended shelf life in greenhouse leafy vegetables.

### 3.9 Seasonality: temperature, light level, and relative air humidity

**Figure 2:**
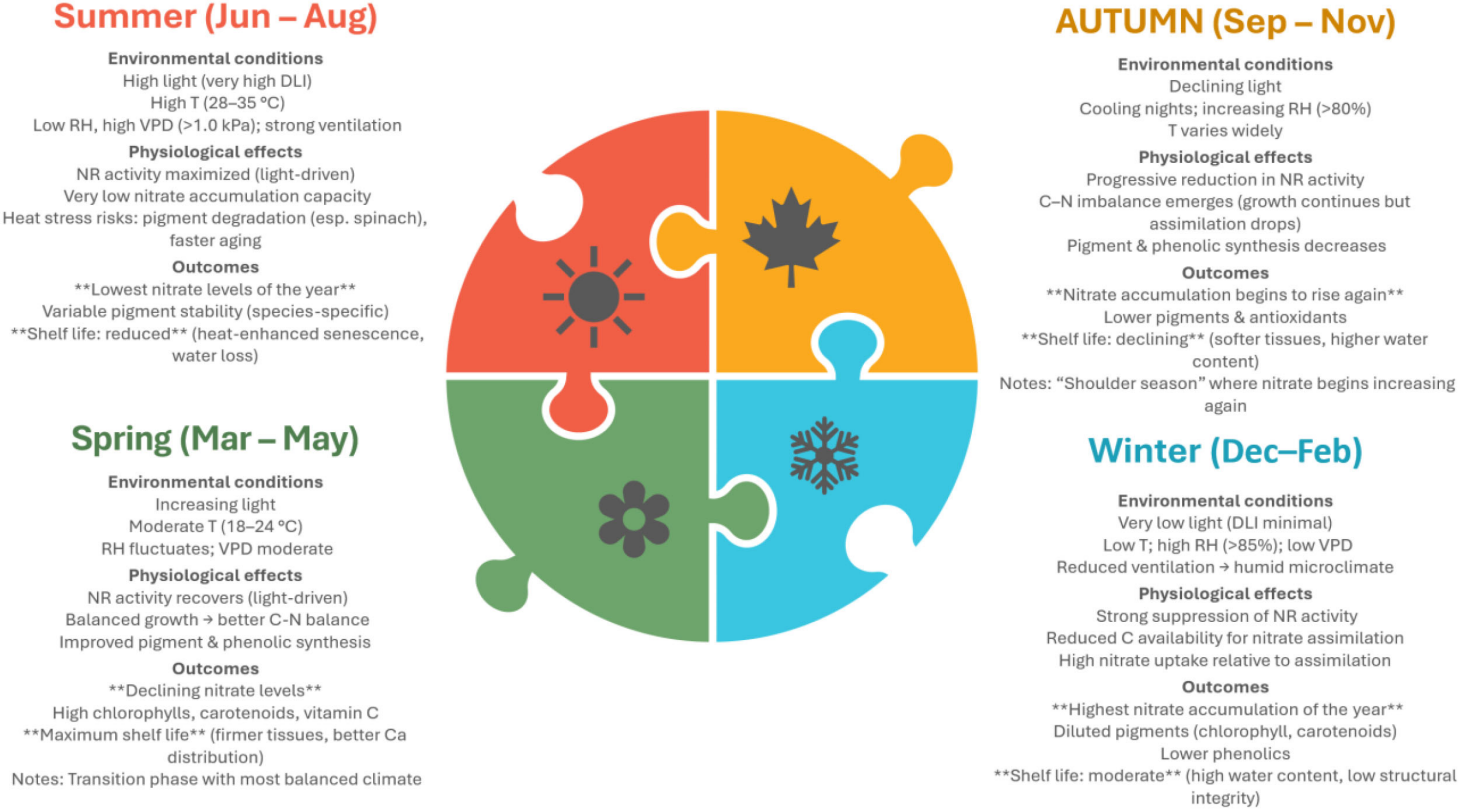
Seasonal dynamics of environmental drivers, nitrate accumulation, and quality outcomes in greenhouse-grown leafy vegetables. C, carbon; Ca, calcium; CO_2_, carbon dioxide; DIF, day–night temperature difference; DLI, daily light integral; DM, dry matter; EC, electrical conductivity; K, potassium; N, nitrogen; RH, relative air humidity; Si, silicon; T, temperature; vitamin C, ascorbic acid; VPD, vapor pressure deficit.

Seasonality imposes predictable yet impactful fluctuations in temperature, light availability, and RH within greenhouses, even under controlled conditions (Flavin Hodge et al., 2018; Gruda et al., 2025). These seasonal changes influence the balance between nitrate uptake and reduction, pigment biosynthesis, antioxidant capacity, and tissue structure (Viršilė et al., 2018; Fanourakis et al., 2025a; Gruda et al., 2025). Winter cropping typically involves low light and cooler temperatures, conditions that slow nitrate assimilation and favor nitrate accumulation (Flavin Hodge et al., 2018; Karavidas et al., 2019; Bian et al., 2020; Gruda et al., 2025). In contrast, summer conditions promote faster growth, higher respiration, and increased WC, but may limit pigment and phenolic synthesis if temperatures become excessive (Fanourakis et al., 2025a; Gruda et al., 2025). Because lettuce, spinach, and rocket differ in thermal preferences and light requirements, seasonal patterns exert species-specific effects on both nutritional quality and shelf life (**Figure 2**).

In lettuce, winter cultivation commonly results in elevated nitrate concentrations due to limited light and reduced NR activity (**Table 3**) (Escobar-Gutiérrez et al., 2002; Bian et al., 2020; Gruda et al., 2025). Although cooler temperatures increase firmness and slow physiological deterioration, the combination of low light and high RH can lead to pale coloration and higher susceptibility to postharvest browning (Kays and Paull, 2004; Martínez-Ispizua et al., 2022a; Salehinia et al., 2024). Spring and autumn conditions generally produce lettuce with better color, balanced nitrate levels, and improved texture, supported by more favorable light–temperature relationships (Dapoigny et al., 2000; Escobar-Gutiérrez et al., 2002; Gruda et al., 2025). Summer cultivation, particularly under warm nights, accelerates leaf expansion and respiration, yielding more succulent tissues with reduced storage potential unless light is abundant and RH is well controlled (Dapoigny et al., 2000; Kays and Paull, 2004; Gruda et al., 2025).

Spinach responds favorably to the cool temperatures and moderate RH characteristic of winter and early spring, which preserve chlorophyll, folates, and ascorbic acid while supporting firm tissue structure (**Table 4**) (Hu et al., 2004; Proietti et al., 2009; Mudau et al., 2019). However, low winter light can increase nitrate concentration and predispose leaves to faster yellowing if antioxidant capacity is insufficient (Bian et al., 2020; Han et al., 2023; Gulyás et al., 2025). During summer, high temperatures and high radiation can accelerate senescence, reduce pigment retention, and increase susceptibility to microbial spoilage (Kays and Paull, 2004; Mudau et al., 2019; Gruda et al., 2025). Spring and autumn typically provide the optimal balance of temperature and light, producing spinach with strong pigmentation, moderate nitrate levels, and longer shelf life (Proietti et al., 2009; Mudau et al., 2019; Gulyás et al., 2025).

Rocket is highly sensitive to seasonal microclimate shifts due to its rapid growth, high nitrate-uptake capacity, and delicate leaf structure (**Tables 5**, **6**) (Hall et al., 2013; Cavaiuolo and Ferrante, 2014; Gruda et al., 2025). In winter, low light markedly increases nitrate accumulation while reducing glucosinolate and phenolic synthesis, resulting in milder flavor and lower antioxidant density; however, cooler temperatures can improve firmness and slow deterioration (Kays and Paull, 2004; Cavaiuolo and Ferrante, 2014; Bian et al., 2020). Spring and autumn typically provide the most favorable balance, adequate light for pungency and phenolic production, moderate temperatures for structural integrity, and overall improved shelf life (Kays and Paull, 2004; Hall et al., 2013; Cavaiuolo and Ferrante, 2014). Summer conditions often impose combined heat and radiation stress: elevated temperatures accelerate growth but restrict NR activity, producing very high nitrate levels (Cavaiuolo and Ferrante, 2014; Bian et al., 2020; Gruda et al., 2025). High respiration rates and softer, water-rich tissues further shorten summer shelf life unless cooling, shading, and RH control are applied (Kays and Paull, 2004; Hall et al., 2013; Gruda et al., 2025).

**Figure 3:**
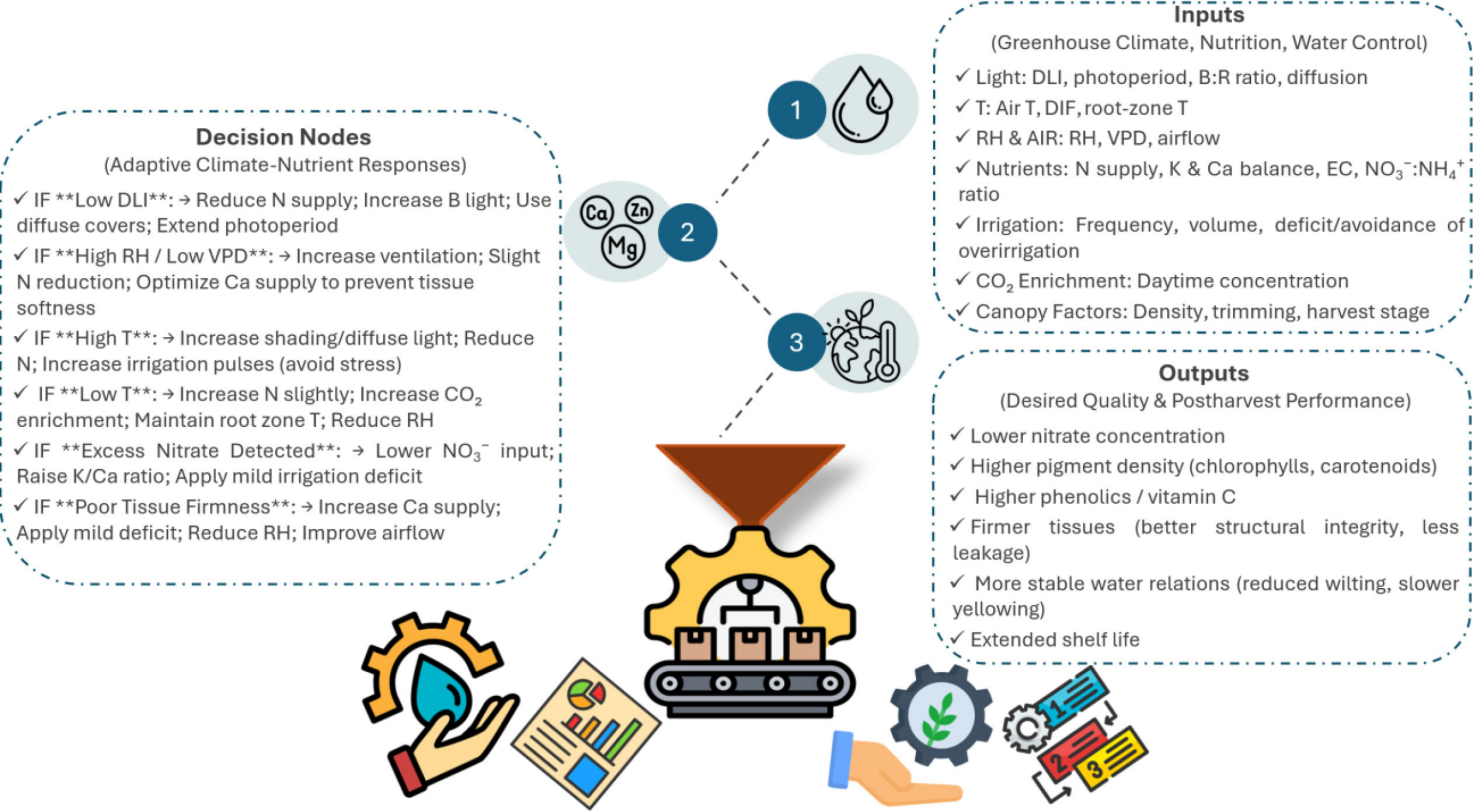
Integrated climate–nutrient–water management model for achieving low nitrate concentration and extended shelf life in greenhouse-grown lettuce, spinach, and rocket. Key environmental and fertigation inputs (top) feed into sequential decision nodes (middle), enabling adaptive adjustments in nitrogen (N) supply, light quality, relative air humidity (RH) control, temperature (T) management, and irrigation. These coordinated responses produce the desired quality outcomes (bottom), including reduced nitrate levels, enhanced pigments and antioxidants, improved tissue firmness, and prolonged shelf life. B, blue; Ca, calcium; CO_2_, carbon dioxide; DIF, day–night temperature difference; DLI, daily light integral; DM, dry matter; EC, electrical conductivity; K, potassium; N, nitrogen; NH_4_^+^, ammonium; NO_3_^−^, nitrate; RH, relative air humidity; Si, silicon; T, temperature; vitamin C, ascorbic acid; VPD, vapor pressure deficit.

Across all three leafy vegetables, seasonality exerts significant influence on nitrate metabolism, pigment and antioxidant profiles, and postharvest behavior. Winter production tends to increase nitrate accumulation and reduce pigment density due to light limitation, whereas summer production risks producing overly succulent leaves with reduced storage potential. Transitional seasons, spring and autumn, typically produce the most balanced nutritional and structural traits. Effective seasonal greenhouse management therefore requires adjusting climate strategies, fertigation practices, and light supplementation according to species-specific physiological needs to maintain consistently high-quality leafy vegetables. These coordinated adjustments are synthesized in the integrated climate–nutrient–water management model presented in **Figure 3**.

### 3.10 Cover materials and shading strategies

Cover materials and shading strategies influence the greenhouse microclimate by altering light transmission, spectral distribution, heat load, and the degree of light diffusion (Baille et al., 2001; Li et al., 2014). These variables shape photosynthetic carbon gain, leaf temperature, transpiration, and pigment and phenolic synthesis, all of which interact with nitrate metabolism and tissue structure (Baille et al., 2001; Bian et al., 2020; Gruda et al., 2025). Diffuse-light–enhancing materials improve canopy light uniformity, reducing self-shading and promoting more balanced nitrate reduction throughout the canopy (Li et al., 2014; Bian et al., 2020). Shading is often applied to mitigate excessive solar radiation and high temperatures but can reduce the DLI and weaken nutritional quality if overused (Baille et al., 2001; Bian et al., 2020; Jung et al., 2024). Species differ in their sensitivity to diffuse light, total irradiance, and shading intensity, leading to distinct impacts on nitrate concentration and postharvest behavior.

In lettuce, cover materials that increase light diffusion improve color uniformity, enhance nitrate reduction in inner leaves, and promote higher phenolic and pigment concentrations (**Table 3**) (Stutte, 2009; Oh et al., 2010; Li et al., 2014). Moderate shading during high-radiation periods prevents heat-induced softening and leaf edge damage while maintaining acceptable light levels for pigment synthesis (Zhao and Carey, 2009). However, excessive shading, especially in already low-light seasons, elevates nitrate accumulation, dilutes chlorophyll and carotenoids, and yields softer tissues with reduced postharvest longevity (Becker et al., 2015). Clear covers provide maximal light transmission but can raise leaf temperatures and RH unless ventilation is optimized (Kim et al., 2022).

Spinach benefits markedly from diffuse light, which enhances pigment retention, increases antioxidant content, and reduces intra-canopy nitrate variability (**Table 4**) (Di Mola et al., 2020; Martínez-Moreno et al., 2024). The crop tolerates moderate shading during periods of high radiation, as spinach maintains pigment density under somewhat reduced DLI (Gao et al., 2020). Nonetheless, excessive shading diminishes chlorophyll concentration, reduces folate and ascorbic acid levels, and raises nitrate content due to limited NR activity (Proietti et al., 2004; Semenova et al., 2023; Martínez-Moreno et al., 2024; Skabelund et al., 2025). Shading combined with inadequate ventilation may increase RH around leaves and predispose spinach to faster postharvest yellowing and microbial spoilage (Conte et al., 2008; Medina et al., 2012; Batziakas et al., 2020). Diffuse covers are particularly effective in maintaining nutritional quality during transitional seasons (Mola et al., 2021; Vuillermet et al., 2023).

Rocket responds very strongly to light diffusion and shading because its glucosinolate- and phenolic-based metabolism depends heavily on sufficient irradiance (**Tables 5**, **6**) (Bell and Wagstaff, 2014; Bell et al., 2020; Mola et al., 2022). Diffuse-light covers enhance light penetration in the canopy, improving pungency, antioxidant capacity, and nitrate-reduction efficiency (Hemming et al., 2008; Bell and Wagstaff, 2014; Mola et al., 2022). In summer, moderate shading helps mitigate heat load, protecting pigments and structural integrity (Poorter et al., 2009; Frary, 2015). However, excessive shading sharply reduces glucosinolate and phenolic synthesis, weakens flavor intensity, and leads to substantial increases in nitrate accumulation (Santamaria, 2006; Bell and Wagstaff, 2014; Caruso et al., 2020). Under heavy shade, rocket typically develops paler, thinner, and more succulent leaves with lower antioxidant density and reduced postharvest performance (Kader, 2002; Poorter et al., 2009; Caruso et al., 2020).

Overall, the choice of cover materials and the application of shading have significant implications for nitrate accumulation, pigmentation, and postharvest performance in lettuce, spinach, and rocket. Diffuse-light–enhancing covers consistently promote more uniform quality and lower nitrate levels, while moderate shading can prevent heat-related quality losses. However, excessive shading compromises nutritional density and accelerates postharvest deterioration. Optimizing light diffusion and shading requires balancing the need for adequate irradiance with the necessity of controlling canopy temperatures, tailoring strategies to the physiological characteristics of each species.

### 3.11 Biostimulants and mineral fortification

Biostimulants and mineral fortification strategies encompass a wide array of substances (e.g., microbial inoculants, seaweed extracts, humic and fulvic acids, amino acids, protein hydrolysates, silicon, and calcium supplements) that enhance plant vigor, nutrient uptake and nitrate assimilation, stress tolerance, and postharvest performance (White and Broadley, 2009; Du Jardin, 2015; Rouphael and Colla, 2020). In leafy vegetables, these products can influence nitrate accumulation by improving nitrogen-use efficiency, stimulating NR activity, or modifying carbon–nitrogen balance (Campbell, 1999; Santamaria, 2006; Voutsinos-Frantzis et al., 2024; Ntanasi et al., 2025). Mineral fortification, particularly with calcium and silicon, strengthens cell walls and membranes, improving firmness, reducing electrolyte leakage, and enhancing shelf life (Kader, 2002; Ma and Yamaji, 2006; White and Broadley, 2009). The response to biostimulants is strongly species-specific, depending on inherent metabolic traits, leaf anatomy, and nutrient uptake characteristics.

In lettuce, biostimulants that enhance nitrate assimilation (e.g., microbial inoculants, amino-acid–based products, or humic substances) can improve nitrate reduction and reduce nitrate accumulation when nitrogen supply is not excessive (**Table 3**) (Atero-Calvo et al., 2025; Madjar et al., 2025; Ntanasi et al., 2025). Seaweed extracts and protein hydrolysates often increase phenolic content, chlorophyll concentration, and antioxidant capacity, contributing to improved nutritional quality and better color retention after harvest (Rouphael et al., 2018; Mughunth et al., 2024; Pantović et al., 2025). Calcium and silicon fortification strengthen leaf structure, reducing susceptibility to mechanical injury, browning, and water-soaked lesions (Kader, 2002; White, 2003; Ma and Yamaji, 2006). However, biostimulants that excessively stimulate growth may inadvertently increase nitrate if metabolic demand is outpaced by uptake, particularly in low-light conditions (Campbell, 1999; Santamaria, 2006; Du Jardin, 2015; Voutsinos-Frantzis et al., 2024).

Spinach responds favorably to biostimulants that promote root activity and antioxidant pathways (**Table 4**) (Rouphael et al., 2018; Rouphael and Colla, 2020; Bonasia et al., 2021). Treatments such as seaweed extracts, amino acids, or microbial consortia (multi-species microbial inoculants) can enhance chlorophyll, lutein, ascorbic acid, and phenolic content, supporting improved oxidative protection and delayed senescence (Fan et al., 2011; Rouphael et al., 2018). Some biostimulants also support more efficient nitrate reduction, moderating nitrate accumulation under high nitrogen input (Di Mola et al., 2020; Saraylou et al., 2022). Mineral fortification, particularly calcium, increases tissue robustness and slows yellowing after harvest (Kader, 2002; White, 2003). Silicon supplementation contributes to improved membrane stability and reduced electrolyte leakage, especially under mild abiotic stress (Ma and Yamaji, 2006; Guntzer et al., 2012; Khosa et al., 2023). However, overapplication of growth-promoting products may lead to softer, more succulent leaves with shorter shelf life (Kader, 2002; White, 2003; Poorter et al., 2009).

Rocket shows strong and rapid responses to biostimulants because of its fast growth and highly active secondary-metabolite pathways (**Tables 5**, **6**) (Bell and Wagstaff, 2014; Bell et al., 2020; El-Nakhel et al., 2023). Elicitors (e.g., seaweed extracts, protein hydrolysates, and microbial preparations) commonly enhance glucosinolate and phenolic synthesis, improving pungency and antioxidant capacity (Bell and Wagstaff, 2014; Bell et al., 2020; Di Mola et al., 2023; El-Nakhel et al., 2023). Several biostimulants also support more efficient nitrate reduction, particularly when nitrogen supply is balanced rather than excessive (Ciriello et al., 2024; Voutsinos-Frantzis et al., 2024; Tallarita et al., 2025). Calcium and silicon fortification markedly improve membrane and cell-wall integrity, critical in this thin-leaf species, reducing electrolyte leakage, delaying wilting, and lowering susceptibility to microbial decay (Kader, 2002; White, 2003; Ma and Yamaji, 2006). Given rocket’s inherently short shelf life, biostimulants that enhance tissue firmness or boost antioxidant potential can produce meaningful improvements in postharvest performance when integrated with appropriate light, VPD, and fertigation management (Kader, 2002; Hodges et al., 2004; Bell et al., 2020; Di Mola et al., 2023; El-Nakhel et al., 2023).

Overall, biostimulants and mineral fortification offer valuable tools for improving nitrate metabolism, nutritional composition, and postharvest performance in greenhouse-grown leafy vegetables. Their benefits are maximized when applied in coordination with balanced nutrient management and appropriate light and temperature conditions. While biostimulants can enhance pigment density, phenolic content, and stress resilience, mineral supplements (such as calcium and silicon) strengthen structural integrity, improving shelf life. Species-specific tailoring of biostimulant and mineral strategies is essential to ensure consistent, high-quality production of lettuce, spinach, and rocket.

### 3.12 Pest and disease pressure and residue-free strategies affecting quality

Pest and disease pressure influences plant physiology, nutrient allocation, and metabolic balance, all of which contribute to nutritional quality and nitrate dynamics in leafy vegetables (Santamaria, 2006; Huber et al., 2012; Bian et al., 2020; Limami and Hirel, 2023). Mild biotic stress often stimulates defense pathways, increasing phenolic and antioxidant synthesis, whereas severe infestations reduce photosynthetic efficiency, nutrient uptake, and tissue integrity (Pérez-Bueno et al., 2019; Iqbal et al., 2021; Saini et al., 2024). Disease lesions or insect feeding damage also elevate respiration and accelerate quality decline after harvest (Al-Khayri et al., 2023; Ufitinema et al., 2024). Residue-free protection strategies (e.g., biological control, plant-defense elicitors, and physical or cultural methods) play a central role in ensuring food safety while supporting plant metabolic homeostasis (Köhl et al., 2019; Akhtar et al., 2024). Species differ substantially in susceptibility to pests and pathogens, physiological responses to elicitation, and the impact of biotic stress on nitrate accumulation and shelf life.

In lettuce, common pests and pathogens such as aphids, thrips, downy mildew, and leaf spot pathogens can significantly affect photosynthetic performance and nitrate assimilation (**Table 3**) (Dai et al., 2009; Prokopová et al., 2010; Nietupski et al., 2022). Mild disease pressure or the application of resistance elicitors may increase phenolic content and antioxidant capacity, modestly improving nutritional quality (Baenas et al., 2014; Xu and Wang, 2025). However, severe infections impair chloroplast function, diminish pigmentation, and may reduce nitrate reduction capacity, leading to higher nitrate accumulation (Misra et al., 2011; Lu and Yao, 2018; Buoso et al., 2019). Tissue damage from pests increases water loss and predisposes leaves to wilting and browning after harvest (Agüero et al., 2011b; Lufu et al., 2020; Brouwer et al., 2023). Biological control and residue-free integrated pest management (IPM) stabilize the physiological condition of lettuce, reducing decay and improving shelf life relative to chemically treated but physiologically stressed crops (Lufu et al., 2020; Lawal et al., 2025).

Spinach is highly susceptible to downy mildew, leaf miners, and certain fungal pathogens (**Table 4**) (Correll, 1994; Mou, 2008; Kandel et al., 2019). Mild elicitation of defense pathways may enhance phenolic compounds and antioxidant levels, supporting improved oxidative protection (Singh, 2016, 2023). However, once infection becomes severe, nitrate assimilation is inhibited due to reduced light interception and impaired metabolic activity (Fagard et al., 2014; Lu and Yao, 2018). Diseased leaves exhibit faster yellowing, increased respiration, and greater microbial susceptibility after harvest (Allende et al., 2004; Kandel et al., 2019). Residue-free IPM approaches (e.g., biocontrol agents, resistant cultivars, and improved ventilation) help maintain pigment integrity and reduce postharvest decay while preserving consumer safety by minimizing pesticide residues (Lawal et al., 2025).

Rocket responds very rapidly to pest and disease pressure because its thin leaves, high respiration rate, and delicate epidermis make it highly vulnerable to tissue disruption (**Tables 5**, **6**) (Siomos and Koukounaras, 2007; Bell et al., 2020; Pane et al., 2021). Aphids, flea beetles, and foliar pathogens quickly reduce photosynthetic capacity and can suppress glucosinolate and phenolic synthesis, diminishing both flavor intensity and antioxidant potential (Soroka and Grenkow, 2013; Neugart et al., 2020; Nietupski et al., 2022; Gebretsadik et al., 2025). Mild elicitation may stimulate glucosinolate production, but significant pest or disease damage increases water loss, electrolyte leakage, and microbial susceptibility, leading to extremely rapid postharvest quality decline (Siomos and Koukounaras, 2007; Frezza et al., 2010; Spadafora et al., 2019; Bell et al., 2020). Effective IPM, based on biological control, clean planting material, and practices that prevent excessive canopy RH, is essential, as even minor tissue injury in rocket can markedly accelerate wilting, electrolyte leakage, and loss of marketable quality (Zhou et al., 2024; Lawal et al., 2025).

Overall, pest and disease pressure interacts strongly with nitrate metabolism, nutritional composition, and postharvest performance in greenhouse-grown leafy vegetables. Mild elicitation may enhance certain quality traits, but significant biotic stress consistently reduces metabolic efficiency, undermines nitrate reduction, and accelerates deterioration. Residue-free IPM strategies improve physiological performance, reduce decay, and preserve the integrity of pigments and antioxidants. Integrating pest management with environmental control is therefore essential for ensuring high-quality production of lettuce, spinach, and rocket.

### 3.13 Major interactions between climate factors

The interaction of climate variables (i.e., temperature, light, CO_2_, RH, VPD, air movement, and nutrient availability) determines the overall physiological environment experienced by greenhouse-grown leafy vegetables (Boulard, 2006; Stanghellini, 2014; Gruda et al., 2025). These factors rarely operate in isolation; instead, they act synergistically or antagonistically to shape nitrate metabolism, pigment synthesis, secondary-metabolite formation, water balance, and tissue structure (Reshi et al., 2023; Fanourakis et al., 2025a, 2025b; Gruda et al., 2025). The combined effects of low light and high nitrogen, high temperature and high RH, or elevated CO_2_ and insufficient irradiance often produce stronger responses than any single factor alone (Stitt, 1999; Frary, 2015; Fu et al., 2017; Abdelhakim et al., 2022; Gruda et al., 2025). Understanding these interactions is essential for predicting quality outcomes and optimizing greenhouse climate strategies for lettuce, spinach, and rocket.

In lettuce, a common and impactful interaction occurs between low light and high nitrogen supply, which results in disproportionately elevated nitrate levels due to limited NR activity (**Table 3**) (Blom-Zandstra, 1989; Stagnari et al., 2015; Seifu, 2017; Bian et al., 2020). High temperature combined with high RH exacerbates leaf succulence, reduces structural integrity, and increases susceptibility to browning, even when light is adequate (Niu and Xiang, 2018; Ifeduba et al., 2024). Conversely, elevated CO_2_ improves pigment formation and nitrate reduction only when light is sufficient; under low light, CO_2_ primarily accelerates growth without enhancing quality (Leakey et al., 2009; Thompson et al., 2017; Wang et al., 2025). Moderate VPD and optimal nitrogen balance interact positively with light diffusion to produce lettuce with uniform color, lower nitrate concentration, and improved shelf life (Cozzolino et al., 2020; Fanourakis et al., 2025a; Gruda et al., 2025).

Spinach displays strong interactive responses among temperature, light, and RH (**Table 4**) (Santamaria, 2006; Monteith and Unsworth, 2013; Frary, 2015; Gent, 2016a; Fu et al., 2017). Cool temperatures and adequate light synergistically enhance chlorophyll, folate, and ascorbic acid accumulation while supporting efficient nitrate reduction (Proietti et al., 2004, 2009; Gent, 2016a; Ramezani et al., 2023; Gulyás et al., 2025). However, warm temperatures paired with low light substantially weaken pigment retention, promote nitrate accumulation, and accelerate postharvest yellowing (Yamauchi and Watada, 1991; Proietti et al., 2004; Mudau et al., 2015; Bian et al., 2020). High RH combined with dense canopy structure increases the risk of microbial contamination and spoilage, even if other conditions are favorable (El-Ramady et al., 2015; Schmitz et al., 2019; Agustin-Salazar et al., 2024; Cheruiyot and Mechesso, 2025). Elevated CO_2_ enhances tissue density and antioxidant potential, but these benefits diminish under light-limited or heat-stressed conditions (Dong et al., 2018; Endo et al., 2022; Huang et al., 2025). Thus, spinach quality depends on balanced coordination of environmental factors.

Rocket is highly sensitive to interactions among nitrogen supply, light quality, temperature, and RH, with combined effects often exceeding the influence of individual factors (**Tables 5**, **6**) (Omirou et al., 2012; Cavaiuolo and Ferrante, 2014; Bell et al., 2020; Gioia et al., 2020; Stajnko et al., 2022). High nitrogen coupled with low light produces extremely high nitrate accumulation while suppressing glucosinolate and phenolic synthesis, leading to weakened flavor and reduced antioxidant potential (Cavaiuolo and Ferrante, 2014; Bian et al., 2020; Kołton, 2022). These interaction effects are consistently stronger in *Eruca* (salad rocket), which shows more extreme nitrate accumulation and faster tissue deterioration under combined low light, high nitrogen, and high RH, whereas *Diplotaxis* (wild rocket) exhibits similar trends but with noticeably lower nitrate rise and better structural resilience (Frezza et al., 2010; Omirou et al., 2012; Cavaiuolo and Ferrante, 2014; Bonasia, 2019; Bian et al., 2020; Signore et al., 2020). Heat stress together with high RH sharply accelerates wilting, electrolyte leakage, and microbial susceptibility, especially in the absence of adequate air movement, resulting in some of the fastest shelf-life declines among leafy vegetables (Agüero et al., 2011b; Bian et al., 2020; Inoue et al., 2021; Palumbo et al., 2024). B or UV-A light can partially offset these negative outcomes by enhancing secondary-metabolite pathways, but only when nitrogen supply, temperature, and VPD remain within optimal ranges (Neugart et al., 2020; Lee et al., 2021; Gruda et al., 2025). Because rocket has inherently high respiration and very rapid turnover, adverse climate-factor interactions manifest more quickly and intensely than in lettuce or spinach, making precise environmental coordination essential for maintaining quality and minimizing nitrate accumulation (Siomos and Koukounaras, 2007; Rogers, 2008; Bonasia, 2019; Bian et al., 2020).

Overall, the interactions among climate variables exert stronger influence on nitrate accumulation, nutritional quality, and postharvest performance than any single environmental factor. Balanced integration of temperature, light, CO_2_, RH, and fertigation is essential to achieve consistent quality across seasons and greenhouse systems. Failure to align these parameters can lead to undesirable outcomes such as excessive nitrate, pigment degradation, and shortened shelf life. A systems-level climate strategy, tailored to the physiological characteristics of each species, is therefore critical for optimizing the quality of greenhouse-grown lettuce, spinach, and rocket.

### 3.14 Climate change implications

Climate change is expected to alter greenhouse production conditions through higher mean temperatures, more frequent heatwaves, altered light regimes, increased RH variability, and greater incidence of extreme weather events affecting ventilation and cooling capacity (Fanourakis et al., 2025b). These changes will modify the physiological balance between growth, nitrate assimilation, pigment formation, and overall leaf structure in leafy vegetables (Bian, 2018; Bian et al., 2020). Rising CO_2_ levels may enhance carbon assimilation but interact with elevated temperatures and lower light availability in complex and sometimes counterproductive ways (Morison and Lawlor, 1999; Zhu et al., 2023; Ainsworth et al., 2025). Given the sensitivity of nitrate metabolism and shelf-life determinants to shifts in temperature, light, and RH, climate change poses significant challenges to achieving consistent quality in greenhouse lettuce, spinach, and rocket.

Lettuce is particularly vulnerable to climate warming because temperatures above the optimal range suppress nitrate reduction, increase nitrate accumulation, and promote softer, more succulent leaves that deteriorate rapidly after harvest (**Table 3**) (Dapoigny et al., 2000; Konstantopoulou et al., 2010; Cometti et al., 2011; Bian et al., 2020). Climate-driven increases in night temperatures may accelerate respiration, reduce pigment retention, and increase browning susceptibility (Jeong, 2015; Kyeong and Lee, 2025; Nitu et al., 2025). More frequent heat events may also increase the risk of tip burn, particularly under high RH, due to impaired calcium transport to rapidly expanding leaf tissue (Barta and Tibbitts, 1991; Moosavi-Nezhad, 2025; Persons et al., 2025). Reduced winter light availability, a projected outcome in some regions due to increased cloudiness, would exacerbate nitrate accumulation and weaken color intensity (Seifu, 2017; Bian et al., 2020; Yegu, 2025). These trends indicate that lettuce quality will increasingly depend on improved temperature control, adaptive shading, and supplemental lighting.

Spinach, although more tolerant to cool conditions, is negatively affected by heat stress, which reduces chlorophyll retention, accelerates senescence, and may impair nitrate reduction under rapid growth (**Table 4**) (Tang et al., 2007; Li et al., 2019; Khan et al., 2026). Climate change is likely to shorten the duration of the cool-season window traditionally used for high-quality spinach production, pushing more production into warmer periods that favor yellowing, nutrient dilution, and microbial spoilage (Castro-Ibáñez et al., 2015; Mudau et al., 2015; Deveci et al., 2019). Increased RH and reduced air movement during extreme weather conditions may intensify disease pressure, further compromising pigment retention and postharvest longevity (Medina et al., 2012; Tudela, 2017). Elevated CO_2_ may partially counteract some quality losses by improving DM content, but only when temperatures and light remain within favorable limits (Proietti et al., 2013; Gil, 2016; Dong et al., 2018).

Rocket is exceptionally vulnerable to climate-change–related increases in temperature and RH because of its high respiration rate, thin leaves, and rapid turnover (**Tables 5**, **6**) (Siomos and Koukounaras, 2007; Amaro et al., 2015; Bonasia, 2019; Romano et al., 2022). Heatwaves accelerate leaf expansion but strongly reduce NR activity, leading to very high nitrate accumulation alongside declines in glucosinolate and phenolic synthesis (Martínez-Ballesta et al., 2013; Cavaiuolo and Ferrante, 2014; Neugart et al., 2020). When elevated temperatures coincide with high RH, wilting, electrolyte leakage, and microbial decay intensify dramatically, resulting in extremely short shelf life (Agüero et al., 2011b; Schmitz et al., 2019). Rising night temperatures are particularly problematic, as they further stimulate respiration and accelerate postharvest deterioration (Siomos and Koukounaras, 2007; Umeohia and Olapade, 2024). Climate-driven shifts in light quality or reduced winter irradiance may weaken flavor intensity by suppressing secondary-metabolite pathways (Pasini et al., 2011; Stajnko et al., 2022; Mainos et al., 2023). Although elevated CO_2_ can increase biomass and leaf thickness, it does not reliably counteract heat-induced quality losses (Chitarra et al., 2015; Romano et al., 2022; Alotaibi, 2025). As a result, future rocket production will depend heavily on improved greenhouse cooling, dynamic shading strategies, and precise RH and air-movement control to maintain both nitrate reduction efficiency and postharvest performance (Siomos and Koukounaras, 2007; Caruso et al., 2020; Guffanti et al., 2022).

In summary, climate change poses substantial risks to nitrate management, nutritional quality, and postharvest performance in lettuce, spinach, and rocket. Rising temperatures, increased RH variability, and altered light environments will intensify existing challenges and reduce the margin for error in greenhouse climate control. While elevated CO_2_ may offer limited compensatory benefits, these advantages are contingent on maintaining favorable temperature and light conditions. Adapting greenhouse production to climate change will require integrated strategies that combine more sophisticated cooling and dehumidification systems, improved shading and lighting control, optimized fertigation regimes, and cultivar selection tailored to emerging climatic realities.

### 3.15 Cultivar differences and genetic adaptation potential

Cultivar selection strongly influences nitrate accumulation, pigment density, secondary-metabolite profiles, and postharvest performance in leafy vegetables (Hall et al., 2015; Bian et al., 2020; Gruda et al., 2025). Genetic differences determine the intrinsic capacity for nitrate uptake and reduction, antioxidant synthesis, structural firmness, and resistance to physiological and microbial disorders (Rouphael et al., 2012; Carillo and Rouphael, 2022; Martínez-Ispizua et al., 2022b). As climate variability increases and the demand for residue-free production intensifies, breeding programs are placing greater emphasis on efficient nitrogen use, robustness under suboptimal environmental conditions, and superior postharvest behavior (Lammerts van Bueren and Struik, 2017; Pagnotta, 2025). Lettuce, spinach, and rocket exhibit substantial intraspecific variability in these traits, offering opportunities for targeted genetic improvement.

In lettuce, cultivars differ widely in nitrate accumulation, leaf structure, pigment intensity, and susceptibility to physiological disorders such as tip burn and pinking (**Table 3**) (Lucarini et al., 2012; Boros et al., 2020; Birlanga et al., 2021; Beacham et al., 2023). Varieties with inherently higher NR activity and greater DM content tend to accumulate less nitrate, especially under low-light conditions (Blom-Zandstra, 1989; Dapoigny et al., 2000; Burns et al., 2011; Stagnari et al., 2015; Bian et al., 2016; Signore et al., 2020). Leaf morphology plays a critical role: crisphead and romaine types often display firmer structure and better shelf life than softer butterhead types, though they may require higher light levels to maintain pigment density (Wien and Stutzel, 1997; Woltering and Witkowska, 2016; Chang et al., 2020). Breeding efforts increasingly target improved calcium use efficiency, reduced browning susceptibility, and enhanced pigment and phenolic profiles to support both nutritional quality and shelf-life performance (Kaushik et al., 2015; Damerum et al., 2020; Weiss and Gruda, 2025).

Spinach shows considerable genetic variability in pigment retention, antioxidant capacity, nitrate-use efficiency, and susceptibility to yellowing (**Table 4**) (Barker et al., 1974; Howard et al., 2002; Yosefi et al., 2010). Certain cultivars maintain higher chlorophyll and ascorbic acid concentrations, resulting in stronger color retention and slower senescence during storage. Some germplasm exhibits improved NR capacity, allowing for lower nitrate accumulation even under reduced light or higher nitrogen supply (Hodges et al., 2004; Spinardi et al., 2010; Dewhirst et al., 2017). Resistance to downy mildew, a major breeding focus, indirectly supports postharvest quality by maintaining leaf integrity, turgor, and metabolic function, and by reducing microbial load (Kandel et al., 2019; Bhattarai et al., 2022). Selective breeding for robust leaf structure and enhanced folate retention is gaining priority in efforts to improve nutritional quality (Garg and Sharma, 2014; Geetha et al., 2022).

Rocket shows substantial cultivar-level variation in nitrate accumulation, glucosinolate and phenolic profiles, flavor intensity, and postharvest durability (**Table 5** and **6**) (Ferrante et al., 2003; Siomos and Koukounaras, 2007; Pasini et al., 2012; Cavaiuolo and Ferrante, 2014; Bell et al., 2017b). *Diplotaxis* (wild rocket) generally contains higher glucosinolate and phenolic concentrations and exhibits stronger antioxidant capacity than *Eruca* (salad rocket), contributing to sharper flavor and slightly greater oxidative protection (Di Gioia et al., 2018; Bell and Wagstaff, 2019; Kurina et al., 2025). However, several *Eruca* (salad rocket) cultivars demonstrate lower nitrate accumulation under balanced nitrogen supply, offering advantages when regulatory limits must be met (Nicola et al., 2005; Bell et al., 2017a; Signore et al., 2020). Cultivars differ markedly in leaf thickness, cuticle robustness, and respiration rate, traits that strongly influence firmness, susceptibility to electrolyte leakage, and overall shelf life (Rogers, 2012; Watkins and Nock, 2012; Shezi et al., 2024). These differences highlight opportunities for genetic improvement, with emerging breeding priorities including enhanced nitrogen-use efficiency, more stable pungency under variable light conditions, and improved tolerance to heat, RH, and other climate-related stresses (Pasini et al., 2012; Bell et al., 2015, 2017b; Signore et al., 2020; Bhattarai et al., 2025; Upadhyay et al., 2025).

Overall, cultivar differences substantially affect nitrate metabolism, nutritional composition, and postharvest performance in greenhouse-grown lettuce, spinach, and rocket. Genetic improvement and informed cultivar choice can mitigate the negative effects of suboptimal environmental conditions and reduce the need for corrective interventions during production. Breeding programs focusing on nitrogen-use efficiency, stress resilience, and postharvest robustness will play a pivotal role in sustaining high-quality leafy vegetable production under increasingly variable greenhouse environments.

## 4 Comparison across environmental factors: relative importance and direction of effects

Considering all preharvest environmental variables, a clear hierarchy emerges in greenhouse-grown lettuce, spinach, and rocket (**Table 7**). Light intensity (or DLI) and air temperature exert the strongest and most direct influence on nitrate accumulation, pigment formation, antioxidant density, and postharvest performance. Adequate light drives photosynthesis and provides the reductants required for nitrate assimilation and secondary-metabolite biosynthesis. Thus, the light environment exerts a positive influence on nutritional quality only when maintained within moderate, well-distributed ranges, whereas either deficiency or excess reduces quality. Moderate temperatures sustain enzymatic activity, pigment biosynthesis, and structural integrity while limiting respiration. Temperature is therefore best interpreted through the lens of metabolic balance: moderate warmth promotes quality, whereas sustained heat stress or deep cold exerts a negative influence on both nutritional attributes and postharvest performance.

**Table 7:** Relative importance and direction of major environmental factors influencing nitrate accumulation, nutritional quality, and shelf life in greenhouse-grown lettuce, spinach, and rocket.

Environmental factor	Relative importance	Predominant direction of effect	Mechanistic basis	Overall influence on nitrate, nutritional quality, and shelf life	Key references
Light intensity & spectral quality	Very high	Moderate–high light → ↓ NO_3_^−^, ↑ pigments, ↑ phenolics; low light → ↑ NO_3_^−^, ↓ antioxidants	Drives photosynthesis, NR activity, pigment/phenolic synthesis	Strongest determinant of NO_3_^−^ levels, color, antioxidant density, and shelf-life performance	(Neely et al., 2010; Rogers, 2012; Bell et al., 2015; Bian et al., 2020; Signore et al., 2020; Min et al., 2021)
Air T (including DIF)	Very high	Moderate T → ↑ NR, ↑ firmness; high T → ↑ NO_3_^−^, ↑ respiration; low T → slow growth	Controls metabolic rate, respiration, NO_3_^−^ reduction, tissue structure	Major driver of NO_3_^−^ accumulation, firmness, and postharvest longevity	(Samuolienė et al., 2009; Sakamoto and Suzuki, 2015; Bian et al., 2020; Gruda et al., 2025)
RH/VPD/airflow	High	Moderate VPD → ↑ firmness, ↓ NO_3_^−^ variability; high RH → ↑ succulence, ↑ NO_3_^−^; high VPD → stress	Regulates transpiration, Ca transport, cuticle formation	Determines leaf firmness, water balance, and risk of decay or wilting	(Amitrano et al., 2019, 2021, 2022, 2025; Medina et al., 2019; Inoue et al., 2021; Yu et al., 2023)
Irrigation regime & water status	High	Mild deficit → ↑ DM, ↑ firmness, ↓ NO_3_^−^ per FW; excess irrigation → ↑ NO_3_^−^ dilution, ↓ firmness	Modulates osmotic balance, leaf succulence, mineral concentration	Strong influence on NO_3_^−^ concentration (via dilution), structural integrity, and shelf life	(Malejane et al., 2018; Abd–Elrahman et al., 2022; Lee et al., 2022; Montazar et al., 2025)
Fertigation (N/K/Ca, EC, salinity)	High	High N → ↑ NO_3_^−^; balanced N with K/Ca → ↓ NO_3_^−^, ↑ antioxidants; moderate EC → ↑ DM	Controls NO_3_^−^ uptake vs reduction, cell-wall strength, pigment formation	Key factor controlling NO_3_^−^ accumulation and structural resilience	(Blom-Zandstra, 1989; Abd–Elrahman et al., 2022; Rubio-Casal and Ibrahim, 2024; Yasin et al., 2025)
CO_2_ enrichment	Moderate	Positive only with adequate light; insufficient light → minimal effect on NO_3_^−^	Enhances C assimilation and DM, alters C:N ratio	Improves tissue density and modestly extends shelf life when conditions are optimal	(Proietti et al., 2013; Qiu et al., 2014; Dong et al., 2018; Esmaili et al., 2020; Li et al., 2020)
Seasonality (T, light, RH)	Moderate	Winter (low light) → ↑ NO_3_^−^; Summer (high T) → ↓ firmness, ↓ pigments	Integrates seasonal fluctuations in light and T	Explains annual variation in NO_3_^−^ levels, coloration, and storage potential	(Campbell, 1999; Kader, 2002; Frary, 2015; Bonan, 2016; Colla et al., 2018)
Cover materials & shading	Moderate	Diffuse light → ↓ NO_3_^−^, ↑ pigments; heavy shading → ↑ NO_3_^−^, ↓ antioxidants	Modifies light uniformity, leaf T, stress load	Improves consistency and reduces heat stress when optimized	(Hemming et al., 2008; Von Zabeltitz, 2011; Li et al., 2014; Frary, 2015; Colla et al., 2018)
Biostimulants & mineral fortification (Ca, Si)	Moderate	Generally positive: ↑ phenolics, ↑ antioxidant activity, ↑ firmness	Enhances nutrient use efficiency, membrane stability, defense pathways	Supports structural integrity and reduces NO_3_^−^ under balanced nutrition	(Santamaria et al., 2001; White, 2003; Ma and Yamaji, 2006; Du Jardin, 2015; Colla et al., 2018; Rouphael and Colla, 2020)
Pest/disease pressure & residue-free IPM	Low–moderate	Mild elicitation → ↑ phenolics; severe stress → ↓ NR, ↑ decay	Affects photosynthesis, induces defense metabolism	Influences decay incidence and postharvest deterioration	(Dixon and Paiva, 1995; Mcdonald et al., 1997; Rosenzweig et al., 2001; Kader, 2002; Huber et al., 2012; Frary, 2015)
Interactions among climate factors	—	Synergistic under balanced regimes; detrimental under extremes	Cross-effects of light × T × RH × N on NO_3_^−^ and pigments	Ultimately determines effectiveness of integrated climate strategies	(Campbell, 1999; Von Zabeltitz, 2011; Monteith and Unsworth, 2013; Frary, 2015; Colla et al., 2018)

Ca, calcium; CO_2_, carbon dioxide; DM, dry matter; EC, electrical conductivity; K, potassium; N, nitrogen; NO_3_^−^, nitrate; NR, nitrate reductase; RH, relative air humidity; Si, silicon; T, temperature; VPD, vapor pressure deficit. Arrows indicate direction of projected change (↑ increase, ↓ decrease).

RH, its functional derivative (VPD), and irrigation/fertigation regimes follow in importance, primarily shaping tissue structure, water balance, nutrient transport, and metabolic efficiency. RH exerts a biphasic effect, beneficial when moderate but clearly detrimental at either extreme. Moderate VPD and RH support balanced growth and calcium delivery, while extremes induce succulent or dehydrated tissues prone to electrolyte leakage, wilting, and microbial colonization. Mild water deficits can enhance DM content and antioxidant accumulation, whereas overirrigation or severe stress compromises tissue integrity and storage resilience. Irrigation effects are therefore non-linear, being beneficial under mild deficit but detrimental under both severe stress and excess. Balanced nutrient supply, particularly of nitrogen, potassium, and calcium, is crucial for nitrate reduction, pigment density, and tissue firmness, whereas excessive nitrogen or EC extremes reduce quality. Proper fertigation therefore exerts a consistently positive influence on both nitrate management and postharvest performance.

CO_2_ enrichment, shading strategies, and biostimulants exert moderate influence, primarily refining rather than determining quality outcomes. CO_2_ enrichment thus exerts a positive but context-dependent effect. Proper CO_2_ enrichment enhances photosynthesis and DM accumulation, diffuse shading improves canopy uniformity and pigment retention, but shading is beneficial only when calibrated precisely. Biostimulants (e.g., calcium, silicon, microbial inoculants) strengthen membranes and delay senescence. Pest and disease pressure, although less influential on nitrate metabolism, strongly affects decay incidence and marketability (Fanourakis et al., 2026). However, mild elicitation through biological control can increase phenolic levels and strengthen defensive barriers.

Seasonal trade-offs in leaf quality are best explained by interactions among light, temperature, and VPD, rather than by season per se. Overall, optimal nutritional quality and postharvest performance are achieved not by maximizing individual factors, but by harmonizing multiple environmental parameters to maintain balanced light interception, temperature, water status, and nutrient supply. This integrative perspective provides a mechanistic basis for precision greenhouse management aimed at consistent production, low nitrate accumulation, high nutritional value, and enhanced postharvest durability.

## 5 Species differences

### 5.1 Environmental sensitivities and quality outcomes

Lettuce, spinach, and rocket differ markedly in leaf anatomy, metabolic profiles, and nitrate-assimilation characteristics (**Table 2**), conferring distinct sensitivities to preharvest environmental conditions (**Table 8**). These species-specific differences shape trade-offs among nitrate accumulation, nutritional quality, and postharvest performance.

**Table 8:** Comparative sensitivity of lettuce, spinach, and rocket to major preharvest environmental factors affecting nitrate accumulation, nutritional quality, and shelf life in greenhouse cultivation.

Environmental factor	Lettuce	Spinach	*Eruca sativa* (salad rocket)	*Diplotaxis tenuifolia* (wild rocket)
Light intensity and quality	Very high influence on NO_3_^−^ reduction, pigments, phenolics; low light → ↑ NO_3_^−^	High influence on chlorophyll retention and antioxidants; B/UV-A → ↑ phenolics	Extremely high influence; low light → very high NO_3_^−^, ↓ pungency; B/UV-A → ↑ glucosinolates	High influence; low light → ↑ NO_3_^−^ but less than *Eruca*; B/UV-A → strong ↑ phenolics & glucosinolates
T (air and leaf)	High; warm T → ↑ NO_3_^−^ & softening; cool T → firmness but pale leaves	Very high; warm T accelerates yellowing and senescence	High; heat → ↑ NO_3_^−^, ↓ glucosinolates, rapid wilting	Moderate–high; more heat-tolerant than *Eruca*; heat still ↑ NO_3_^−^ but pigments more stable
RH/VPD	Very high; high RH → soft tissues, ↑ NO_3_^−^; moderate VPD optimal	High; high RH → faster microbial spoilage	Very high; high RH → extreme succulence, electrolyte leakage, fast decay	High; still sensitive but more resilient; high RH → ↑ WC but slower quality loss than *Eruca*
CO_2_ enrichment	Moderate; beneficial only under adequate light	Moderate; ↑ DM and pigments when DLI sufficient	Moderate; ↑ biomass; NO_3_^−^ reduction improves only with high light	Moderate; ↑ DM and phenolics; better integration with light than *Eruca*
Irrigation regime/water status	High; mild deficit → ↑ firmness, phenolics; excess → ↑ succulence, ↑ NO_3_^−^	High; moderate deficit → ↑ DM; excess → fragile leaves	Very high; both deficit and excess rapidly increase deterioration	High; mild deficit tolerated better; severe deficit still → ↓ shelf life
Fertigation (N/K/Ca, EC)	Very high; high N → ↑ NO_3_^−^; Ca essential for tip-burn control; moderate EC beneficial	High; balanced N/K/Ca → ↑ pigments, ↓ NO_3_^−^	Very high; high N → extremely high NO_3_^−^; K & S crucial for flavor profile	High; high N → ↑ NO_3_^−^ (less extreme than *Eruca*); moderate EC → ↑ phenolics & glucosinolates
Seasonality (light, T, RH)	Strong seasonal NO_3_^−^ variability; winter → ↑ NO_3_^−^	Strong; winter deficits → ↑ NO_3_^−^, ↑ yellowing risk	High seasonal sensitivity; winter → ↑ NO_3_^−^, ↓ pungency; summer heat → bolting and rapid softening	High but more stable; winter → ↑ NO_3_^−^, modest ↓ pungency; summer → moderate heat stress
Cover materials & shading	High; diffuse light → ↓ NO_3_^−^; heavy shading → ↑ NO_3_^−^	Moderate; diffusive films stabilize pigments	Very high; shading strongly affects glucosinolates, pungency & NO_3_^−^	High; diffuse covers beneficial; heavy shading → ↓ flavor but less effect on NO_3_^−^ than *Eruca*
Biostimulants/mineral fortification (Ca, Si)	Moderate–high; ↑ firmness, ↓ browning	High; ↑ antioxidants, ↑ pigment retention	High; elicitors strongly ↑ glucosinolates & phenolics, ↓ leakage	High; similar response but more stable retention during storage
Pest/disease pressure & IPM	Moderate; infections ↑ browning & ↓ pigments	High; downy mildew drastically reduces quality	Very high; even slight tissue damage → rapid decay	High; still vulnerable but slower deterioration than *Eruca*
Overall sensitivity pattern	Highly sensitive to RH/VPD, low light, Ca deficiency	Sensitive to heat and RH; cool-adapted	Extremely sensitive to low light, nutrient imbalance, and RH; shortest shelf life	Sensitive but more resilient; better pigment/stress retention; longer shelf life than *Eruca*

Ascorbic acid, vitamin C; B, blue (spectra); Ca, calcium; CO_2_, carbon dioxide; DM, dry matter; EC, electrical conductivity; K, potassium; N, nitrogen; NO_3_^−^, nitrate; NR, nitrate reductase; RH, relative air humidity; Si, silicon; T, temperature; UV, ultraviolet; VPD, vapor pressure deficit. Arrows indicate direction of projected change (↑ increase, ↓ decrease).

Lettuce is highly sensitive to RH fluctuations and calcium-related disorders, owing to its thin and poorly developed cuticle, high WC, and comparatively low NR activity. Even moderate deviations in light, RH, or nutrient supply can elevate nitrate levels and produce soft, succulent tissues prone to electrolyte leakage, wilting, and rapid postharvest deterioration.

Spinach, in contrast, benefits from thick leaf blades and high chlorophyll density, combined with cool-adapted physiology, conferring resilience to low temperatures and high photosynthetic efficiency under moderate light, thereby supporting effective nitrate reduction. However, it is highly susceptible to temperature-driven pigment degradation and accelerated senescence, particularly under warm conditions or high RH, which reduces chlorophyll retention and promotes microbial spoilage regardless of nitrate status. Biostimulants, calcium supplementation, and balanced nitrogen/potassium fertigation can support pigment retention and improve postharvest behavior, but even small deviations in growth conditions accelerate quality loss.

Rocket exhibits the strongest dependence on light and nutrient balance, reflecting its thin leaf structure, rapid biomass accumulation, and highly active glucosinolate–phenolic metabolism. It accumulates nitrate readily under low light or high nitrogen supply, and minor deviations in light, RH, or nutrient inputs can rapidly compromise flavor intensity, antioxidant content, and postharvest performance. Its delicate tissues are highly prone to wilting, electrolyte leakage, and microbial decay immediately after harvest.

Collectively, lettuce shows the greatest sensitivity to RH and calcium-related disorders, spinach to temperature-induced senescence, and rocket to light and nutrient balance for maintaining low nitrate and high antioxidant traits. These distinctions confirm that no single environmental strategy is suitable for all leafy vegetables. Instead, greenhouse management must target species-specific physiological constraints: precise light and VPD optimization for lettuce, stable cool conditions with RH moderation for spinach, and balanced nitrogen–light inputs with tight VPD control for rocket. The unique interplay between structural resilience and metabolic flexibility defines each species’ capacity to maintain nutritional quality and postharvest performance under greenhouse conditions.

### 5.2 Physiological mechanisms linking environment, metabolism, and quality formation

Environmental conditions regulate nitrate accumulation and the synthesis of other quality-related metabolites in leafy vegetables through interconnected physiological and biochemical processes (Santamaria, 2006; Colla et al., 2018). Central to these processes is the balance between carbon assimilation and nitrogen metabolism, which determines whether absorbed nitrate is reduced and incorporated into organic compounds or accumulated in leaf tissues (Stitt, 1999; Masclaux-Daubresse et al., 2010; Marschner, 2012). Nitrate reduction requires metabolic energy and reducing power generated during photosynthesis, as well as carbon skeletons required for amino-acid synthesis (Stitt, 1999; Marschner, 2012; Masclaux-Daubresse et al., 2010). Consequently, environmental conditions that enhance photosynthetic activity, particularly adequate light availability, moderate temperature, and balanced nutrient supply, generally promote nitrate assimilation and reduce nitrate accumulation (Santamaria, 2006; Lillo, 2008; Colla et al., 2018).

Light plays a particularly important regulatory role because it simultaneously controls photosynthetic carbon supply and the activity of key enzymes involved in nitrogen metabolism, including NR and nitrite reductase (NiR), which catalyzes the subsequent reduction of nitrite to ammonium during nitrate assimilation (Stitt, 1999; Lillo, 2008; Marschner, 2012). Under high irradiance, increased carbohydrate production supports nitrate reduction and the synthesis of amino acids and proteins (Stitt, 1999; Lillo, 2008; Marschner, 2012). In contrast, under low-light conditions or when carbon availability becomes limiting, nitrate uptake may exceed the metabolic capacity for reduction, resulting in nitrate accumulation within leaf vacuoles (Stitt, 1999; Lillo, 2008; Marschner, 2012).

Beyond nitrogen metabolism, environmental factors also influence the biosynthesis of secondary metabolites that determine the nutritional and functional quality of leafy vegetables. Phenolics, flavonoids, glucosinolates, and antioxidant compounds are frequently regulated through carbon allocation and redox signaling pathways associated with photosynthetic activity and environmental stress (Akula and Ravishankar, 2011; Neilson et al., 2013). Hormonal signaling pathways, including cytokinins, abscisic acid, and jasmonates, also interact with environmental cues to regulate nitrogen metabolism, stress responses, and the synthesis of defense-related metabolites (Wasternack and Hause, 2013; Kiba and Krapp, 2016; Kazan, 2015). Moderate abiotic stresses, such as elevated irradiance, mild water limitation, or balanced mineral nutrition, can stimulate antioxidant metabolism, whereas excessive nitrogen availability or strong shading often promotes rapid biomass accumulation but dilutes these compounds through growth-related dilution effects (Akula and Ravishankar, 2011; Neilson et al., 2013; Colla et al., 2018).

Source–sink dynamics further modulate these responses by influencing how assimilated carbon and nitrogen are partitioned among growth, storage, and defense metabolism (Herms and Mattson, 1992; Neilson et al., 2013). In addition, structural and anatomical traits, including leaf thickness, cuticle development, stomatal density, and tissue WC, affect transpiration, internal gas exchange, and metabolite stability (Poorter et al., 2009; Lawson and Blatt, 2014; Marschner, 2012). Together, these physiological mechanisms explain how greenhouse environmental conditions shape not only nitrate levels but also the broader nutritional composition and postharvest performance of leafy vegetables (**Figure 1**).

### 5.3 Physiological and anatomical mechanisms underlying postharvest deterioration

While Section 5.2 addressed the physiological mechanisms through which environmental factors influence metabolite accumulation before harvest, the following section focuses on the physiological and anatomical processes governing postharvest tissue stability and quality deterioration.

Species-specific differences in postharvest behavior ultimately arise from a set of interacting physiological and anatomical mechanisms that determine tissue integrity, metabolic resilience, and the rate of quality loss after harvest (Kader, 2002; Wang et al., 2018b; Lara et al., 2019). While environmental drivers and compositional traits (**Tables 2**, **8**) describe what differs among species, these mechanisms explain why lettuce, spinach, and rocket show distinct shelf-life trajectories.

A central mechanism underlying postharvest deterioration is the initial metabolic status of the harvested tissue. Leaves harvested with high concentrations of pigments (e.g., chlorophylls and carotenoids), antioxidants (e.g., ascorbic acid and phenolics), and structural carbohydrates require longer postharvest periods before these compounds decline below sensory or functional thresholds (Kader, 2002; Toivonen and Brummell, 2008; Gil, 2016). For example, high initial chlorophyll content delays the visual onset of yellowing simply because a larger pigment pool must be degraded before discoloration becomes perceptible (Kader, 2002; Malka et al., 2026). Similarly, high antioxidant capacity slows oxidative damage to membranes and proteins, thereby reducing the rate of senescence-related processes (e.g., lipid peroxidation, enzyme activation, and electrolyte leakage) (Hodges et al., 2004; Gill and Tuteja, 2010; Sharma et al., 2012).

In contrast, tissues harvested with low DM content and high WC are intrinsically more prone to rapid deterioration. Low DM implies lower concentrations of sugars, starch, and structural compounds per unit fresh weight, resulting in limited metabolic buffering capacity after harvest (Marcelis et al., 1998; Kays and Paull, 2004; Saltveit, 2019). Such tissues exhibit higher respiration rates per unit DM, faster depletion of soluble carbohydrates, and reduced energy availability for maintenance metabolism (Paul and Foyer, 2001; Frary, 2015; Saltveit, 2019). This combination accelerates senescence, promotes membrane destabilization, and increases susceptibility to microbial growth (Hodges et al., 2004; Lukatkin et al., 2012; Sharma et al., 2012; Saltveit, 2019; Lawal et al., 2025). The frequently observed “watery” texture of rocket and some lettuce types therefore not only reflects sensory attributes but represents a physiological state characterized by low carbon density and weak structural integrity.

Soluble sugars and starch play a particularly important role as postharvest energy substrates. After harvest, photosynthesis ceases, and respiration relies entirely on stored carbohydrates (Kader, 2002; Frary, 2015; Saltveit, 2019). Leaves with higher sugar and starch reserves can sustain cellular homeostasis for longer periods, delaying chlorophyll breakdown, protein degradation, and loss of membrane functionality (Wingler et al., 2006; Zeeman et al., 2010; Thomas and Ougham, 2014; Saltveit, 2019). Conversely, tissues with limited carbohydrate pools experience rapid respiratory exhaustion, leading to accelerated pigment loss, increased electrolyte leakage, and faster collapse of sensory quality (Brouquisse et al., 1998; Wingler et al., 2006; Thomas and Ougham, 2014; Saltveit, 2019). This mechanism partly explains why environmental conditions that promote higher DM accumulation (e.g., high light, moderate VPD, balanced nutrition) consistently improve shelf life across leafy species.

At the cellular level, membrane lipid composition represents a fundamental determinant of postharvest performance. Membrane stability reflects a balance between fluidity and oxidative vulnerability: higher proportions of unsaturated fatty acids maintain membrane fluidity and reduce chilling injury during cold storage, whereas excessive polyunsaturation increases susceptibility to lipid peroxidation under oxidative stress (Murata and Los, 1997; Hodges et al., 1999; Upchurch, 2008; Sharma et al., 2012). In this context, shelf life depends on the balance between unsaturation, which protects against chilling, and saturation, which confers resistance to oxidative damage (Murata and Los, 1997; Hodges et al., 1999; Upchurch, 2008; Sharma et al., 2012). Preharvest conditions that promote optimal membrane integrity (e.g., adequate calcium and silicon supply, moderate light stress, and strong antioxidant capacity) therefore indirectly extend shelf life by preserving compartmentalization and delaying oxidative breakdown.

Leaf anatomy and cuticle development further modulate these physiological processes. Thicker leaves with well-developed mesophyll layers, lower surface-to-volume ratios, and more substantial cuticles exhibit slower water loss, reduced transpiration-driven stress, and greater resistance to mechanical damage (Kerstiens, 1996; Poorter et al., 2009; Lara et al., 2014; Saltveit, 2019). Spinach, with its relatively thick lamina and robust midrib, therefore retains turgor and structural integrity longer than lettuce or rocket (Kerstiens, 1996; Kader, 2002; Poorter et al., 2009; Saltveit, 2019). By contrast, rocket leaves combine thin epidermal layers, high stomatal density, and limited cuticular protection, creating a structural configuration that favors rapid dehydration, high respiration per unit mass, and extreme postharvest fragility (Kerstiens, 1996; Jin et al., 2009; Poorter et al., 2009; Saltveit, 2019).

In parallel, calcium-mediated cell wall stabilization represents a key structural mechanism underlying tissue firmness and storability. Calcium cross-links pectins in the middle lamella, increasing cell wall rigidity, reducing enzymatic cell separation, and slowing tissue softening (White, 2003; Brummell, 2005; Hepler, 2005; Vicente et al., 2007). Leaves with higher calcium concentrations therefore maintain mechanical integrity for longer periods, whereas limited calcium transport to rapidly expanding leaves (e.g., under high RH and low transpiration) predisposes tissues to early textural collapse, increased electrolyte leakage, and faster quality loss (Saure, 2001; Kader, 2002; White, 2003; Hepler, 2005).

An additional and often overlooked mechanism linking preharvest climate to postharvest behavior is stomatal density and functionality. Growing conditions strongly regulate stomatal development and physiological control, with light intensity, spectral composition, VPD, temperature, and CO_2_ concentration determining both stomatal density and aperture dynamics (Lawson and Blatt, 2014). High light and moderate VPD typically increase stomatal density and conductance, whereas elevated CO_2_ and high RH reduce stomatal density but often impair stomatal responsiveness. These anatomical and functional traits persist after harvest and directly influence transpiration rate, water loss kinetics, and the onset of wilting (Fanourakis et al., 2016, 2020). Leaves with high stomatal density and limited postharvest stomatal closure exhibit accelerated dehydration and faster loss of turgor, whereas tissues with lower stomatal density or more effective stomatal regulation retain water longer and display extended shelf life (Kerstiens, 1996; Kader, 2002; Gil, 2016; Sinha et al., 2018).

Nitrate content may act primarily as an indirect marker of postharvest vulnerability, rather than a direct causal agent. High nitrate accumulation is typically associated with growth conditions characterized by low light, high nitrogen supply, high RH, and rapid tissue expansion ((Blom-Zandstra, 1989; Escobar-Gutiérrez et al., 2002; Santamaria, 2006; Burns et al., 2011). These same conditions also promote low DM content, diluted carbohydrate pools, reduced cuticle development, high stomatal density, and elevated WC (Kerstiens, 1996; Lake et al., 2001; Santamaria, 2006; Poorter et al., 2009; Burns et al., 2011). As a result, high nitrate concentrations frequently co-occur with poor postharvest performance, not because nitrate itself directly accelerates deterioration, but because it reflects a physiological state dominated by succulence, low carbon density, and limited metabolic resilience (Blom-Zandstra, 1989; Kader, 2002; Santamaria, 2006; Poorter et al., 2009; Burns et al., 2011). In this sense, nitrate functions as an integrative indicator of growth conditions that predispose tissues to short shelf life and reduced nutritional quality.

Postharvest behavior is also strongly influenced by the microbial ecology of the leaf surface. Leaf anatomy, cuticle thickness, surface roughness, and the presence of soluble exudates determine microbial attachment and proliferation after harvest (Beuchat, 2002; Gil et al., 2009; Lara et al., 2014). Thin-cuticle leaves with high surface moisture and nutrient loss provide favorable conditions for microbial growth, accelerating visual spoilage, off-odors, and sensory rejection (Beuchat, 2002; Allende et al., 2004; Gil et al., 2009). Species such as rocket, which combine high WC with fragile epidermal barriers, are therefore particularly susceptible to microbially driven deterioration.

Finally, endogenous hormonal balance contributes to species-specific senescence dynamics. Lower cytokinin levels and increased ethylene sensitivity promote chlorophyll degradation, protein catabolism, and membrane breakdown (Grbić and Bleecker, 1995; Hörtensteiner and Kräutler, 2011; Zwack and Rashotte, 2013). Preharvest stress conditions that shift hormonal signaling toward senescence-related pathways therefore predispose leaves to faster postharvest decline, even when visual quality at harvest appears acceptable.

Overall, postharvest deterioration in leafy vegetables can be mechanistically interpreted as a progressive failure of metabolic homeostasis, driven by carbohydrate depletion, oxidative stress, membrane destabilization, microbial proliferation, impaired stomatal regulation, and structural collapse. Environmental conditions that enhance DM accumulation, sugar reserves, pigment pools, membrane integrity, calcium distribution, stomatal functionality, and tissue robustness before harvest therefore create leaves with inherently greater physiological resilience, explaining their superior shelf-life performance across species.

## 6 Methodological notes: experimental design, confounders, and reporting standards

Interpretation of greenhouse studies examining the impact of environmental conditions on nitrate accumulation, nutritional quality, and shelf life in leafy vegetables is frequently constrained by methodological inconsistency. Variation in experimental design, climate control precision, and data reporting often obscures genuine physiological responses and limits comparability across studies. As a result, contradictory findings regarding nitrate dynamics, pigment retention, or postharvest performance often stem more from inconsistent methodologies than from biological variability.

Many studies manipulate a single factor (e.g., such as light intensity, nitrogen supply, or irrigation) while other co-varying climate variables remain unquantified. Yet in greenhouse environments, air temperature, RH, VPD, CO_2_, and radiation are inherently interdependent. Increasing temperature typically lowers RH and raises VPD; supplemental lighting elevates leaf temperature and modifies transpiration; and altering irrigation regimes affects canopy RH and nutrient uptake patterns. Without quantifying these interactive shifts, effects risk being misattributed to the nominal treatment. The frequent use of single-compartment greenhouses without true replication further compounds the problem. Plants within one compartment are often treated as independent replicates, inflating statistical significance and masking spatial or temporal gradients. Robust inference requires independent compartments, replicated seasonal cycles, or split-plot designs that separate biological from environmental replication.

**Figure 4:**
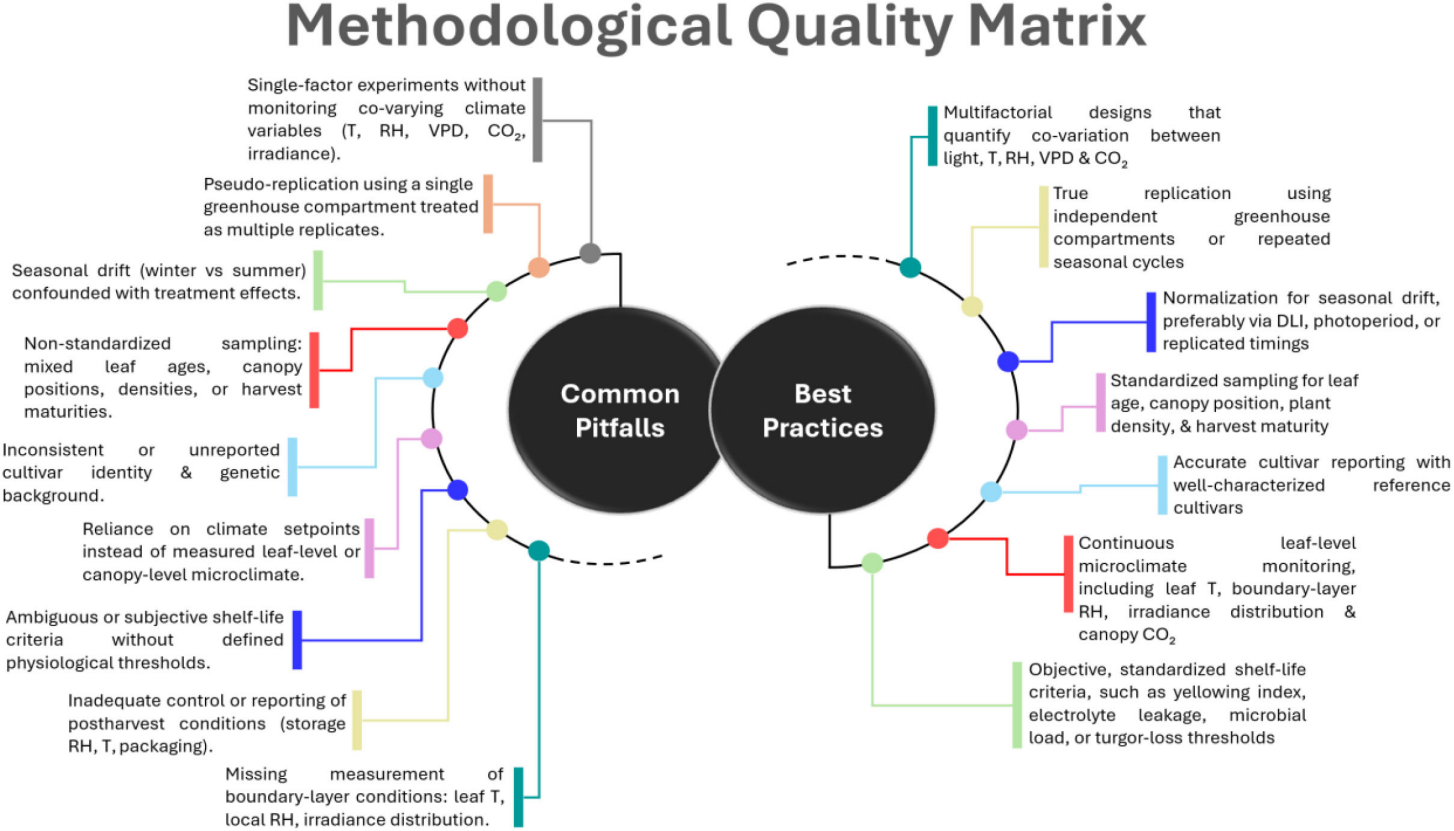
Common methodological pitfalls in greenhouse nitrate–quality studies (left) and recommended best practices for experimental rigor, climate monitoring, and shelf-life standardization (right). CO_2_, carbon dioxide; DIF, day–night temperature difference; DLI, daily light integral; RH, relative air humidity; T, temperature; vitamin C, ascorbic acid; VPD, vapor pressure deficit.

Sampling heterogeneity introduces additional uncertainty. Differences in leaf age, canopy position, harvest timing, and plant density often exceed the magnitude of treatment effects on nitrate concentration or pigment levels. Moreover, cultivar identity strongly modulates nitrate accumulation and metabolic responses. Lettuce, spinach, and rocket cultivars vary widely in nitrate-reductase capacity, leaf structure, and antioxidant potential. Failure to standardize cultivar selection or to report genetic background accurately limits cross-study comparability. Strict maturity criteria, randomized sampling across canopy layers, and the inclusion of well-characterized reference cultivars would substantially reduce bias. A structured overview of these methodological pitfalls and the recommended standards for experimental rigor is presented in **Figure 4**.

Seasonal and temporal variation represent further confounding factors. Experiments conducted at different times of year may attribute differences in nitrate, chlorophyll, or phenolics to treatments rather than to natural changes in DLI, photoperiod, or background microclimate. Replicating key treatments across seasons or normalizing responses to cumulative light exposure is essential to isolate true treatment effects. Similar caution applies to postharvest evaluations: storage temperature, RH, packaging conditions, and handling practices can easily overshadow preharvest influences. Standardizing the definition of “end of shelf life” based on measurable thresholds (e.g., yellowing index, electrolyte leakage, microbial load, or loss of turgor) would greatly improve cross-study comparability, paralleling long-standing recommendations from floriculture vase-life research.

Accurate microclimate monitoring remains a central methodological gap. Many studies report only climate setpoints rather than measured values at leaf level. However, actual microenvironmental conditions (i.e., leaf surface temperature, boundary-layer RH, irradiance distribution, and CO_2_ concentration at canopy height) are the proximal drivers of nitrate assimilation, pigment metabolism, and water-loss kinetics. Continuous logging of both ambient and leaf-level parameters is therefore indispensable. Without these measurements, studies risk drawing conclusions based on assumed rather than experienced microclimates.

Ultimately, methodological rigor is the foundation for meaningful synthesis. Incomplete climate characterization, pseudo-replication, inconsistent sampling, and poorly standardized shelf-life assessments explain much of the divergent evidence surrounding how temperature, light, RH, and nutrition influence nitrate accumulation and storage performance in leafy vegetables. Future research should adopt multifactorial designs that better reflect real greenhouse dynamics, integrate high-resolution climate monitoring with biochemical and postharvest measurements, and ensure open reporting of raw environmental and compositional data. Only through transparent, standardized, and physiologically grounded methodologies can the field progress toward predictive understanding of how greenhouse environments shape the nutritional and postharvest attributes of lettuce, spinach, and rocket.

## 7 Knowledge gaps and research priorities

Despite considerable progress in understanding how greenhouse environments influence nitrate accumulation and nutritional and postharvest attributes in leafy vegetables, current knowledge remains fragmented and unevenly distributed across species, environmental factors, and analytical endpoints. Most available studies describe isolated responses to single variables rather than the integrated behavior of complex greenhouse systems. Consequently, our ability to predict nitrate levels, pigment and antioxidant dynamics, and shelf-life outcomes under fluctuating greenhouse conditions remains limited. Closing these gaps requires both methodological refinement and conceptual expansion in research design.

A primary knowledge gap concerns the quantitative integration of environmental variables. Light intensity, light quality, temperature, RH, VPD, CO_2_ concentration, and nitrogen or water supply interact continuously, yet these interactions are rarely examined systematically in lettuce, spinach, or rocket. Most experiments manipulate one stimulus (e.g., nitrogen supply or light) while assuming constancy in other factors that in reality co-vary. For example, supplemental lighting elevates leaf temperature and alters transpiration; reducing temperature shifts VPD and leaf WC; and adjusting irrigation changes canopy RH and nitrogen uptake. Because these interdependencies are frequently unquantified, the true hierarchy of environmental drivers of nitrate accumulation and shelf life remains uncertain. Multifactorial experiments combined with continuous microclimate monitoring and mechanistic or data-driven modeling are needed to disentangle these relationships.

A second limitation involves the uneven species representation in the literature. Lettuce has received extensive attention, especially regarding nitrate accumulation and light effects, whereas spinach and rocket remain comparatively under-studied, particularly in relation to integrated climate control. The distinct physiological characteristics of these species (i.e., spinach’s cool-adapted metabolism, rocket’s glucosinolate-driven antioxidant system, and their contrasting nitrate-reduction capacities) are insufficiently quantified under controlled environmental variation. Comparative cross-species studies using harmonized protocols would allow broader generalization of responses and facilitate transferability of findings from well-studied models (e.g., lettuce) to less-studied crops (e.g., rocket).

A third gap concerns the weak linkage between preharvest environmental physiology and postharvest performance. Many studies quantify nitrate, chlorophyll, phenolics, or glucosinolates at harvest but do not assess how these translate into shelf-life behavior, including yellowing, wilting, electrolyte leakage, microbial growth, or oxidative degradation. Conversely, postharvest studies often lack detailed preharvest climate records. Integrated designs that track individual leaves or batches from greenhouse microclimate exposure through storage, combined with non-destructive methods (e.g., color metrics, water-status sensors, or optical phenotyping), are essential for establishing causal relationships between environmental conditions and shelf-life outcomes.

Temporal dimensions of environmental responses constitute another unresolved issue. Short-term exposure studies dominate the literature, yet many processes governing nitrate accumulation, pigment formation, and structural robustness are cumulative or stage-dependent. Responses to high VPD or low light, for example, differ markedly between early leaf expansion and late maturation phases. Long-term time-series measurements of leaf temperature, transpiration, NR activity, pigment dynamics, and antioxidative metabolism are needed to identify developmental windows during which environmental optimization yields the greatest gains in quality.

Standardization of analytical metrics presents a further priority. Definitions of “nutritional quality” vary widely: some studies emphasize nitrate content alone, others focus on antioxidants, ascorbic acid, chlorophyll, phenolics, or glucosinolates. Shelf life is variously defined by visual yellowing, weight loss, turgor retention, microbial load, or electrolyte leakage. This lack of uniform benchmarks undermines cross-study synthesis and prevents quantitative meta-analysis. Establishing harmonized protocols, potentially through international working groups or open-access databases, would greatly enhance reproducibility and comparability of results.

The integration of digital technologies and modeling tools remains underutilized in leafy vegetable research. Greenhouse control systems routinely log high-resolution temperature, RH, VPD, radiation, CO_2_, and irrigation data, yet these datasets are rarely linked to nitrate or nutritional measurements. Machine-learning approaches could identify the most influential predictors of nitrate accumulation or yellowing kinetics from large environmental datasets, provided that standardized metadata and sufficient sample sizes are available. Similarly, mechanistic models coupling carbon–nitrogen interactions, water relations, and oxidative metabolism could allow predictive simulations of quality outcomes under different climate-control strategies.

A further frontier concerns genotype by environment interactions. Few studies systematically compare cultivars varying in NR efficiency, leaf thickness, pigment biosynthesis pathways, or postharvest robustness under controlled environmental gradients. Such comparisons could identify genetic traits conferring resilience to low light, high RH, or temperature fluctuations, traits that are increasingly important for modern greenhouse cultivation. Integrating physiological phenotyping with transcriptomic or metabolomic analyses would help elucidate regulatory mechanisms driving nitrate assimilation and nutritional quality.

Finally, sustainability considerations require greater attention. Environmental strategies that enhance quality (e.g., supplemental lighting, CO_2_ enrichment, or high-frequency fertigation) must be evaluated in the context of energy, water, and carbon costs. Life-cycle and techno-economic assessments that incorporate reductions in food loss due to extended shelf life would provide more holistic insight into the sustainability of quality-oriented greenhouse strategies.

In summary, future research on lettuce, spinach, and rocket should pursue five major priorities: (1) multifactorial experiments integrating key environmental variables; (2) standardized analytical and reporting protocols linking preharvest and postharvest phases; (3) continuous, high-resolution monitoring of canopy and leaf-level microclimate; (4) genotype-based investigations identifying resilience traits; and (5) data-driven and mechanistic modeling frameworks predicting nitrate and quality outcomes under dynamic greenhouse conditions.

By addressing these gaps, the field can transition from descriptive studies to predictive, physiology-based design of greenhouse environments that optimize nitrate management, nutritional value, and shelf-life performance. The major methodological gaps and corresponding research priorities are summarized conceptually in **Figure 4**.

## 8 Conclusions

The collective evidence reviewed demonstrates that preharvest environmental conditions in greenhouse production exert profound and multifaceted effects on nitrate accumulation, nutritional quality, and postharvest longevity of leafy vegetables. Among all cultivation variables, light intensity and temperature emerge as the most decisive determinants, governing the balance between carbon assimilation, nitrate reduction, pigment biosynthesis, and metabolic homeostasis. Adequate and well-distributed radiation combined with moderate temperatures promotes efficient NR activity, higher chlorophyll and antioxidant levels, and improved structural integrity. In contrast, low light or excessive heat suppresses nitrate reduction, dilutes pigments and phenolics, and accelerates senescence processes that compromise shelf life. These findings reaffirm that the microclimate experienced by the leaf, particularly leaf-level irradiance, temperature, and boundary-layer RH, rather than nominal air setpoints, is the true driver of final quality.

RH and VPD shape water relations and calcium distribution, thereby regulating leaf texture, resilience to mechanical damage, and susceptibility to postharvest decay. Water and nutrient management serve as physiological modulators of nitrate concentration and leaf robustness. Mild deficit irrigation and balanced nitrogen–potassium–calcium fertigation support lower nitrate accumulation and higher DM content, whereas overirrigation or excessive nitrogen supply increases succulence, elevates nitrate concentration, and reduces postharvest performance. CO_2_ enrichment, diffuse-light covers, and biostimulant or mineral fortification generally exert positive influences by enhancing photosynthesis, reinforcing cellular structure, and stabilizing pigments. Conversely, imbalanced climatic interactions or unmanaged pathogen pressure impose cascading negative effects on both nutritional value and shelf life.

Clear species-specific patterns emerge across the three crops. Lettuce shows the greatest sensitivity to light deficiency and high RH, conditions that elevate nitrate accumulation and predispose tissues to rapid postharvest deterioration. Spinach, while more resilient in nitrate-reduction capacity, is highly susceptible to temperature-induced chlorophyll degradation and accelerated loss of antioxidant compounds, making precise thermal management essential. Rocket displays the strongest dependence on balanced nitrogen supply and adequate light for maintaining low nitrate levels and high glucosinolate and phenolic concentrations, yet its delicate leaf structure renders it vulnerable to RH-related wilting and electrolyte leakage. These distinctions highlight the need for crop-specific environmental optimization rather than uniform greenhouse prescriptions across leafy vegetables.

Despite substantial advances, notable knowledge gaps remain. Most studies treat environmental factors in isolation, neglecting the synergistic and antagonistic interactions that characterize real greenhouse conditions. Limited replication, heterogeneous maturity standards, and incomplete microclimate reporting continue to constrain cross-study comparability and mechanistic interpretation. Future research must adopt integrative, multifactorial experimental designs that trace preharvest microclimate exposure through postharvest evolution, supported by continuous leaf-level monitoring and standardized analytical protocols. Coupling physiological insights with data-driven and mechanistic modeling will enable greenhouse management strategies that optimize nitrate control, nutritional density, and postharvest performance simultaneously.

Ultimately, improving the quality and shelf life of greenhouse-grown lettuce, spinach, and rocket aligns with broader sustainability goals: reducing postharvest losses, minimizing resource inputs, and providing nutritionally dense produce year-round. By refining environmental control based on mechanistic understanding rather than empirical adjustment, next-generation greenhouse systems can function as precision bioreactors, delivering leafy vegetables that meet high standards of nutritional value, food safety, and postharvest performance, while remaining resilient under increasingly variable climatic and economic conditions.

## Supplementary material

Supplementary Table 1 Typical greenhouse nitrate concentration ranges (mg NO_3_^−^ kg^−1^ FW) in additional leafy vegetables. Crops are ranked from lowest to highest nitrate accumulation. B, blue (spectra); DLI, daily light integral; FW, fresh weight; N, nitrogen; NO_3_^−^, nitrate; NR, nitrate reductase; UV-A, ultraviolet A; VPD, vapor pressure deficit.
