# Supplementary material for: Greenhouse climate shapes nitrate levels, nutritional quality, and shelf life in leafy vegetables: species-specific responses
Source: Front Plant Sci. 2026 May 7;17:1806482. doi: 10.3389/fpls.2026.1806482 (PMC13189842; doi:10.3389/fpls.2026.1806482)
Supplement: Supplementary Table 1 — Typical greenhouse nitrate concentration ranges (mg NO3− kg−1 FW) in additional leafy vegetables. Crops are ranked from lowest to highest nitrate accumulation. B, blue (spectra); DLI, daily light integral; FW, fresh weight; N, nitrogen; NO3−, nitrate; NR, nitrate reductase; UV-A, ultraviolet A; VPD, vapor pressure deficit. [file Table1.docx]

**Supplementary Table S1**. Typical greenhouse nitrate concentration ranges (mg NO₃⁻ kg⁻¹ FW) in additional leafy vegetables. Crops are ranked from lowest to highest nitrate accumulation. B, blue (spectra); DLI, daily light integral; FW, fresh weight; N, nitrogen; NO₃⁻, nitrate; NR, nitrate reductase; UV-A, ultraviolet A; VPD, vapor pressure deficit.

| **Crop** | **Typical nitrate range in greenhouse production** | **Notes** | **Key references** |
| --- | --- | --- | --- |
| Kale (*Brassica oleracea* var. *sabellica*) | 300–1500 | Very low accumulator; high NR activity; rich in glucosinolates. | (Santamaria, 2006; EFSA Journal, 2008; Trejo-Téllez et al., 2019; Bian et al., 2020) |
| Moringa leaves (*Moringa oleifera*) | 400–1800 | Efficient NO₃⁻ reduction; high protein and chlorophyll content. | (Makkar and Becker, 1996; Santamaria, 2006; Anwar et al., 2007; Leone et al., 2015; Saini et al., 2016) |
| Broccoli leaves (*Brassica oleracea* var. *italica*) | 500–2000 | Low-to-moderate accumulator; physiologically similar to kale. | (Santamaria, 2006; Cartea and Velasco, 2008; EFSA Journal, 2008; Trejo-Téllez et al., 2019; Bian et al., 2020) |
| Beet greens (*Beta vulgaris* subsp. *vulgaris*) | 800–2500 | Moderate accumulator; often lower than Swiss chard. | (Cantliffe, 1973; Santamaria, 2006; EFSA Journal, 2008; Bian et al., 2020) |
| Mustard greens (*Brassica juncea*) | 1500–3500 | Moderate accumulator; sensitive to VPD and shading. | (Santamaria, 2006; EFSA Journal, 2008; Trejo-Téllez et al., 2019; Bian et al., 2020; Sehgal et al., 2022; Gruda et al., 2025) |
| Mizuna (*Brassica rapa* var. *japonica*) | 1500–4000 | NO₃⁻ rises under high density or low DLI. | (Santamaria, 2006; EFSA Journal, 2008; Kalisz et al., 2012; Bian et al., 2020; Park et al., 2020) |
| Tatsoi (*Brassica rapa* var. *narinosa*) | 2000–4500 | Strong response to light; NO₃⁻ decreases with B/UV-A. | (Santamaria, 2006; EFSA Journal, 2008; Viršilė et al., 2018; Trejo-Téllez et al., 2019; Bian et al., 2020; Li et al., 2021) |
| Pak choi / Bok choy (*Brassica rapa* subsp. *chinensis*) | 2000–5000 | Medium-to-high accumulator; higher NO₃⁻ in baby-leaf stage. | (Santamaria, 2006; Wang et al., 2007; EFSA Journal, 2008; Bian et al., 2020) |
| Endive / Escarole (*Cichorium endivia*) | 2500–5000 | Strong seasonal effect; winter typically >5000 without supplemental light. | (Escobar-Gutiérrez et al., 2002; Santamaria, 2006; EFSA Journal, 2008; Bian et al., 2020; Conversa et al., 2021; Voutsinos-Frantzis et al., 2024; Gruda et al., 2025) |
| Amaranth leaves (*Amaranthus* spp.) | 2500–5500 | Among highest within minor leafy crops; responds strongly to N supply. | (Santamaria, 2006; EFSA Journal, 2008; Onyango et al., 2012; Bian et al., 2020) |
| Swiss chard (*Beta vulgaris* var. cicla) | 2500–6000 | High accumulator; physiology similar to spinach; >7000 under low DLI. | (Cantliffe, 1973; Santamaria, 2006; EFSA Journal, 2008; Bian et al., 2020; Boucher et al., 2023) |
| Malabar spinach (*Basella alba*) | 3000–6000 | High NO₃⁻ under low light; water-rich tissues. | (Santamaria, 2006; EFSA Journal, 2008; Bian et al., 2020; Zhang et al., 2024; Gruda et al., 2025) |
| Water spinach (*Ipomoea aquatica)* | 3000–7000 | Very high NO₃⁻ under shaded greenhouse conditions. | (Santamaria, 2006; EFSA Journal, 2008; Jampeetong et al., 2012; Kitayama et al., 2019) |
| Purslane (*Portulaca oleracea*) | 3500–8000 | One of the highest accumulators; rapidly increases under high N. | (Santamaria, 2006; EFSA Journal, 2008; Bian et al., 2020; Voutsinos-Frantzis et al., 2024; Ntanasi et al., 2025) |
| Celery leaves (*Apium graveolens*) | 4000–8000 | Accumulates NO₃⁻ in petioles and leaves; sensitive to shading. | (Santamaria et al., 1999; Kader, 2002; Santamaria, 2006; EFSA Journal, 2008; Bian et al., 2020) |

**References**

Anwar, F., Latif, S., Ashraf, M., and Gilani, A. H. (2007). Moringa oleifera: a food plant with multiple medicinal uses. Phytother. Res. 21, 17–25. doi: 10.1002/ptr.2023.

Boucher, L., Nguyen, T.-T.-A., Brégard, A., Pepin, S., and Dorais, M. (2023). Optimizing light use efficiency and quality of indoor organically grown leafy greens by using different lighting strategies. Agronomy 13, 2582. doi: 10.3390/agronomy13102582.

Cantliffe, D. J. (1973). Nitrate accumulation in table beets and spinach as affected by nitrogen, phosphorus, and potassium nutrition and light intensity. Agron. J. 65, 563–565. doi: 10.2134/agronj1973.00021962006500040012x.

Cartea, M. E., and Velasco, P. (2008). Glucosinolates in Brassica foods: bioavailability in food and significance for human health. Phytochem. Rev. 7, 213–229. doi: 10.1007/s11101-007-9072-2.

EFSA Journal (2008). Nitrate in vegetables—scientific opinion of the panel on contaminants in the food chain. EFSA J. 6:689, 1–79. doi: 10.2903/j.efsa.2008.689.

Jampeetong, A., Brix, H., and Kantawanichkul, S. (2012). Effects of inorganic nitrogen forms on growth, morphology, nitrogen uptake capacity and nutrient allocation of four tropical aquatic macrophytes (Salvinia cucullata, Ipomoea aquatica, Cyperus involucratus and Vetiveria zizanioides). Aquat. Bot. 97, 10–16. doi: 10.1016/j.aquabot.2011.10.004.

Kalisz, A., Sękara, A., and Kostrzewa, J. (2012). Effect of growing date and cultivar on the morphological parameters and yield of Brassica rapa var. japonica. Acta Sci. Pol. Hortorum Cultus 11, 131–143. ISSN 1644-0692.

Kitayama, M., Nguyen, D. T. P., Lu, N., and Takagaki, M. (2019). Effect of light quality on physiological disorder, growth, and secondary metabolite content of water spinach (Ipomoea aquatica Forsk) cultivated in a closed-type plant production system. Hortic. Sci. Technol. 37, 206–218. doi: 10.12972/kjhst.20190020.

Leone, A., Fiorillo, G., Criscuoli, F., Ravasenghi, S., Santagostini, L., Fico, G., et al. (2015). Nutritional characterization and phenolic profiling of Moringa oleifera leaves grown in Chad, Sahrawi refugee camps, and Haiti. Int. J. Mol. Sci. 16, 18923–18937. doi: 10.3390/ijms160818923.

Li, J., Wu, T., Huang, K., Liu, Y., Liu, M., and Wang, J. (2021). Effect of LED spectrum on the quality and nitrogen metabolism of lettuce under recycled hydroponics. Front. Plant Sci. 12:678197. doi: 10.3389/fpls.2021.678197.

Makkar, H. P. S., and Becker, K. (1996). Nutritional components of extracted Moringa oleifera leaves. Anim. Feed Sci. Technol. 63, 211–228. doi: 10.1016/S0377-8401(96)01023-1.

Onyango, C. M., Harbinson, J., Imungi, J. K., Shibairo, S. S., and Van Kooten, O. (2012). Influence of organic and mineral fertilization on germination, leaf nitrogen, nitrate accumulation and yield of vegetable amaranth. J. Plant Nutr. 35, 342–365. doi: 10.1080/01904167.2012.639917.

Park, C. H., Bong, S. J., Lim, C. J., Kim, J. K., and Park, S. U. (2020). Transcriptome analysis and metabolic profiling of green and red mizuna (Brassica rapa L. var. japonica). Foods 9, 1079. doi: 10.3390/foods9081079.

Saini, R. K., Manoj, P., Shetty, N. P., Srinivasan, K., and Giridhar, P. (2016). Relative bioavailability of folate from the traditional food plant Moringa oleifera L. as evaluated in a rat model. J. Food Sci. Technol. 53, 511–520. doi: 10.1007/s13197-015-1828-x.

Sehgal, A., Reddy, K. R., Walne, C. H., Barickman, T. C., Brazel, S., Chastain, D., et al. (2022). Climate stressors on growth, yield, and functional biochemistry of two Brassica species, kale and mustard. Life 12, 1546. doi: 10.3390/life12101546.

Trejo-Téllez, L. I., Estrada-Ortiz, E., Gómez-Merino, F. C., Becker, C., Krumbein, A., and Schwarz, D. (2019). Flavonoid, nitrate and glucosinolate concentrations in Brassica species are differentially affected by photosynthetically active radiation, phosphate and phosphite. Front. Plant Sci. 10:371. doi: 10.3389/fpls.2019.00371.

Wang, H.-J., Wu, L.-H., Wang, M.-Y., Zhu, Y.-H., Tao, Q.-N., and Zhang, F.-S. (2007). Effects of amino acids replacing nitrate on growth, nitrate accumulation, and macroelement concentrations in Pak-choi (Brassica chinensis L.). Pedosphere 17, 595–600. doi: 10.1016/S1002-0160(07)60070-8.

Zhang, Y., Cheng, W., Di, H., Yang, S., Tian, Y., Tong, Y., et al. (2024). Variation in nutritional components and antioxidant capacity of different cultivars and organs of Basella alba. Plants 13, 892. doi: 10.3390/plants13060892.
